# Distinct Immunological Landscapes of HCMV-Specific T Cells in Bone Marrow and Peripheral Blood

**DOI:** 10.3390/pathogens14080722

**Published:** 2025-07-22

**Authors:** Sarah E. Jackson, Rosie Fairclough, Veronika Romashova, Georgina Okecha, Mark R. Wills

**Affiliations:** Department of Medicine, Cambridge Institute of Therapeutic Immunology and Infectious Disease, Cambridge Biomedical Campus, School of Clinical Medicine, University of Cambridge, Cambridge CB2 2QQ, UK; rf520@cam.ac.uk (R.F.); vr263@cam.ac.uk (V.R.); go203@cam.ac.uk (G.O.)

**Keywords:** human cytomegalovirus (HCMV), bone marrow, peripheral blood, T cells, latency, lytic antigens, checkpoint inhibitory receptors

## Abstract

Human cytomegalovirus (HCMV) establishes lifelong latency in the host, with the bone marrow (BM) CD34+ cells serving as a key reservoir. To investigate tissue-specific immune responses to CMV, we analysed paired peripheral blood mononuclear cells (PBMCs) and bone marrow mononuclear cells (BMMNCs) from HCMV-seropositive donors using multiparametric flow cytometry and cytokine FluroSpot assays. We assessed immune cell composition, memory T cell subsets, cytokine production, cytotoxic potential, activation marker expression, and checkpoint inhibitory receptor (CIR) profiles, both ex vivo and following stimulation with lytic and latent HCMV antigens. BMMNCs were enriched in CD34+ progenitor cells and exhibited distinct T cell memory subset distributions. HCMV-specific responses were compartmentalised: IFN-γ responses predominated in PBMCs following lytic antigen stimulation, while IL-10 and TNF-α responses were more prominent in BMMNCs, particularly in response to latent antigens. US28-specific T cells in the BM showed elevated expression of CD39, PD-1, BTLA, CTLA-4, ICOS, and LAG-3 on CD4+ T cells and increased expression of PD-1, CD39, BTLA, TIGIT, LAG-3, and ICOS on CD8+ T cell populations, suggesting a more immunoregulatory phenotype. These findings highlight functional and phenotypic differences in HCMV-specific T cell responses between blood and bone marrow, underscoring the role of the BM niche in shaping antiviral immunity and maintaining viral latency.

## 1. Introduction

The majority of our current understanding of the specificity and effector functions of HCMV-specific T cells is derived from studies of the peripheral blood compartment. Over the past several decades, extensive analyses have characterised the antigenic specificity and frequency of both CD4+ and CD8+ T cells using whole infected cell lysates and overlapping synthetic peptide pools representing individual HCMV open reading frames (ORFs) (summarised [1]). These studies have employed a range of assays to determine T cell specificity and frequency, including detection of cell surface expressed activation markers, secretion or intracellular production of antiviral cytokines (e.g., IFN-γ and TNF-α), allele-specific MHC class I and II multimers, and cytotoxicity assays. Many of these approaches have been integrated with flowcytometry-based phenotypic markers of, e.g., T cell memory subsets, chemokine receptors and markers of exhaustion, yielding a comprehensive understanding of T cell specificity, frequency, effector function, and memory phenotype (reviewed in [2]).

Recent findings have revealed that cells latently infected with Human Cytomegalovirus (HCMV) express a repertoire of latency-associated proteins, including UL138, LUNA (UL81-82as), US28, UL111A (vIL-10), and UL144 [3,4,5,6,7,8]. Many of these HCMV proteins have a role in the maintenance of latency [9]; however, expression of these proteins could thus make these latent cells targets for T cells. This has led to the identification of both CD4+ and CD8+ T cells specific for these latency-associated antigens. Notably, in addition to classical antiviral CD8+ T cells that express CD107a and secrete IFN-γ and TNF-α, and CD4+ T cells with similar effector profiles, IL-10-secreting CD4+ and CD8+ T cells specific to these latent antigens have also been identified [10,11,12,13].

It is particularly noteworthy that, despite the generation of T cells targeting antigens expressed by latently infected cells, HCMV latency persists throughout life [14]. This persistence suggests that latently infected cells evade immune surveillance. Such immune evasion may be mediated by several mechanisms, including IL-10-driven immunosuppression by HCMV-specific T cells and the influence of the local microenvironment surrounding latently infected cells [15,16,17].

HCMV latency is maintained in specific anatomical niches, with the bone marrow (BM) being of particular interest [18,19]. We have previously demonstrated that latently infected CD34+ stem cells and CD14+ monocytes secrete both viral and cellular IL-10, as well as TGF-β, all of which contribute to the establishment of an immunosuppressive microenvironment [15,20]. Interestingly, the bone marrow has been shown to serve as a reservoir for memory T cell responses to systemic pathogens, including those elicited by vaccines such as Tetanus Toxoid and Measles, Mumps, and Rubella (MMR) [21]. It also harbours memory responses to cytomegalovirus (CMV). Recent studies in humans have demonstrated that, following an MMR booster, bone marrow-resident CD4+ memory T cells can be mobilised into the bloodstream [22], indicating that these cells are capable of responding to antigenic re-challenge and migrating to sites of infection.

Importantly, these studies also show that the maintenance of memory T cells in the bone marrow does not require persistent antigen presence [23], allowing the bone marrow to function as a long-term reservoir for pathogen-specific memory T cells [24]. Mouse models have been instrumental in elucidating how memory T cell populations are established and maintained within the bone marrow microenvironment. Unlike other tissue-resident sites such as mucosal surfaces or skin, the bone marrow is accessible only via the bloodstream, as it lacks lymphatic drainage [25].

Murine studies have revealed that the bone marrow contains specialised niches formed by mesenchymal stromal cells that support interactions with memory immune cells, including plasma cells and memory T cells [26]. In particular, stromal cells that secrete IL-7 and express VCAM-1 are thought to play a key role in the generation and maintenance of memory T helper cells [27,28].

A combination of murine and human studies has identified a set of markers—CD69 and CD25 (in the absence of activation), along with the integrin VLA-2 (CD49b) and CXCR4—that facilitate T cell entry into the bone marrow and their establishment within stromal memory niches [21,25,26,27,29,30,31]. In addition to these bone marrow-specific markers, tissue-resident memory T cells are known to express multiple inhibitory and checkpoint molecules. Regulatory T cells (Tregs) are also preferentially maintained in the bone marrow [23,32,33].

To date, relatively few studies have examined tissue compartments such as lymph nodes, bone marrow, spleen, lungs, and intestines for the presence of HCMV-specific T cells [34]. Investigations of bone marrow–resident CMV-specific T cells have primarily focused on responses to immunodominant lytic antigens such as pp65 and IE [21,35,36,37,38]. Moreover, there is a notable lack of comparative analyses assessing the distribution of CD4+ and CD8+ T cells specific for latency-associated HCMV proteins in bone marrow versus peripheral blood mononuclear cells (PBMCs) from the same donor.

Previously, we analysed PBMC and bone marrow samples—collected at different time points—from a single CMV+ donor, examining T cell responses to peptides representing 12 HCMV proteins, including those expressed during both latent and lytic infection. This preliminary analysis clearly demonstrated the presence of IFN-γ– and IL-10–secreting CD4+ and CD8+ T cells in the bone marrow that responded to a broad range of HCMV proteins [11,34].

Therefore, we sought to determine whether distinct populations of CMV-specific T cells are present in the bone marrow compared to peripheral blood in temporally matched samples. In particular, we aimed to characterise differences in the bone marrow–resident CMV-specific T cell response to latency-associated proteins such as US28, vIL-10, LUNA, and UL138, which are likely to be locally expressed within the bone marrow microenvironment. Furthermore, we compared these responses to those directed against immunodominant lytic CMV proteins—pp65, IE, and gB—present in both bone marrow and peripheral blood compartments.

We demonstrated that comparative analysis of PBMCs and bone marrow mononuclear cells (BMMNCs) revealed distinct immunological profiles. BMMNCs exhibited reduced frequencies of CD4+, CD8+ T cells, NK cells, and B cells but were enriched for CD34+ hematopoietic stem and progenitor cells within the CD45− fraction. Cytokine assays demonstrated compartmentalised T cell responses to HCMV antigens: IFN-γ responses predominated against classic HCMV lytic peptides, while IL-10 and TNF-α responses were more prominent in response to proteins expressed during latency, particularly in BMMNCs, and these responses were largely single cytokine secreters.

Phenotypic profiling showed that BMMNC-derived CD4+ T cells had fewer naïve-like cells and more differentiated subsets, with low ex vivo activation and generally low checkpoint inhibitory receptor (CIR) expression, with the exception of a few CIRs. Notably, CTLA-4 and 2B4 displayed reciprocal expression patterns, and CD2 was broadly expressed. CD8+ T cell analysis revealed similar baseline phenotypes across compartments, but HCMV peptide stimulation induced distinct cytokine and CIR expression profiles. In particular, US28 (latent antigen) elicited stronger IL-10 responses and higher expression of CIRs (e.g., PD-1, CTLA-4, LAG-3) in BMMNCs compared to IE1 (lytic antigen). Antigen-specific responses were associated with distinct memory subsets and CIR expression patterns, suggesting that bone marrow-resident T cells responding to latent HCMV antigens adopt a more immunoregulatory phenotype, potentially reflecting chronic antigen exposure.

## 2. Materials and Methods

### 2.1. Source of Samples and Donor Characteristics

Paired bone marrow and peripheral blood mononuclear cells from three HCMV-seropositive donors were purchased from AllCells (distributed by Lonza, Slough, UK). The age, sex and HLA characteristics of the donors are summarised in Table 1.

### 2.2. Preparation of Frozen Peripheral Blood and Bone Marrow Mononuclear Cells

The frozen bone marrow and peripheral blood mononuclear cells (BMMNC and PBMNC) were removed from liquid nitrogen storage and rapidly warmed, cells were immediately diluted in excess defrosting media (warmed TexMACS (Miltenyi Biotec, Woking, UK)). Cells were washed by centrifugation for 10 min at 300× *g* before being resuspended in TexMACS supplemented with 10 U/mL of the DNase Benzonase (Merck Millipore, Dorset, UK) and incubated for 1 h at 37 °C. Cells were again washed by centrifugation for 10 min at 300× *g* and resuspended in TexMACS and rested at 37 °C overnight prior to use.

### 2.3. HCMV ORF Peptide Libraries

Peptide libraries consisting of 15 mer overlapping by 10 amin acid peptides were synthesised from 12 HCMV ORF encoded proteins (UL138, LUNA (UL81-82as), US28, UL111A (vIL-10), UL83 (pp65), UL144 (with known strain variants included), UL123 (IE1), UL122 (IE2), US3, UL82 (pp71), UL28 and UL55 (gB)) by either ProImmune PEPScreen (Oxford, UK) or JPT Peptide Technologies GmbH (Berlin, Germany as described previously [13]. The individual lyophilized peptides from each ORF library were reconstituted and used as previously described [39].

### 2.4. Major Lymphocyte Subsets Phenotyping Comparison of PBMC with BMMNC

Defrosted and rested overnight paired PBMC and BMMNC samples were washed and then resuspended with FACS wash (DPBS without calcium and magnesium (Merck Millipore) supplemented with 0.5% BSA (Miltenyi Biotec, Woking, UK) and 2 mM EDTA (Thermo Fisher Scientific, Hemel Hempstead, UK) in polypropylene FACS tubes. The resuspended cells were blocked for 10 min with TruStain FcX (BioLegend, San Diego, CA, USA) prior to staining with either the antibody or isotype control mixes, both contained Brilliant Stain Buffer Plus (BD Biosciences, Wokingham, UK), True Stain Monocyte Blocker (BioLegend, San Diego, CA, USA) and LIVE/DEAD Fixable Aqua Dead Stain Kit (Thermo Fisher Scientific, Hemel Hempstead, UK). Antibodies and isotype control antibodies used in this panel are detailed in Table S1. Cells were vortexed with the antibody mixes and incubated for 30 min at 4 °C in the dark and then washed in excess FACS wash. Samples were fixed with Fluorofix Buffer (BioLegend, San Diego, CA, USA) and stored in the dark at 4 °C until acquisition on a 5-laser LSR Fortessa cytometer (BD Biosciences, Wokingham, UK) using FACS Diva software. Fluorescence Minus One Controls and single colour compensation controls (AbC Total Antibody Compensation Bead Kit and ArC Amine Reactive Compensation Bead kit—Thermo Fisher Scientific, Hemel Hempstead, UK) were also prepared and run with the samples. The acquired data was compensated using the Autospill algorithm [40] in FlowJo v10.10.0 (BD Biosciences, Wokingham, UK) transformed and then each FCS file was quality controlled prior to further processing using the PeacoQC plugin [41] in FlowJo. The processed samples were analysed using the isotype and FMO controls to set positive staining gates for the markers. Equal numbers of live PBMC and BMMNC events for all 3 donors were down sampled and concatenated with added keywords to enable dimensionality reduction t-distributed stochastic neighbour embedding (tSNE) visualisation to be performed using the embedded tSNE function in FlowJo [42,43], and a clustering algorithm plugin FlowSOM [44] was also used to analyse the data. The analysis workflow and representative data from the flow cytometry analysis are illustrated in Figure S1.

### 2.5. Triple Fluorospot Assay

Triple FluoroSpot-FLEX kits (human IFNγ, IL-10 and TNFα (Mabtech AB, Nacka Strand, Sweden)) were prepared following the manufacturers instructions. The defrosted and rested paired PBMC and BMMNC samples were added to the plates with 90,000–150,000 cells per well in triplicate of BMMNC and 150,000 cells per well in triplicate of PBMC. The samples were stimulated with HCMV protein peptide pools as described, and with an unstimulated and positive control mix (containing anti-CD3 (Mabtech AB, Nacka Strand, Sweden), Staphylococcus enterotoxin B, phytohemagglutinin, pokeweed mitogen and lipopolysaccharide (all Merck Millipore, Dorset, UK)) for 40 h at 37 °C to maximise the IL-10 signal produced. The cells and media were decanted and the assay developed, the developed plates were read using an AID iSpot Reader (Oxford Biosystems, Oxford, UK) and counted using AID EliSpot v7 software (Autoimmun Diagnostika GmbH, Straberg, Germany) using distinct counting protocols for IFNγ, IL-10 and TNFα secretion. The input CD3+ T cells were enumerated by staining the samples with a mix of CD3-FITC, CD4-PE and CD8-PerCP-Cy5.5 (BioLegend, San Diego, CA, USA) and LIVE/DEAD Fixable Far Red Dead Stain Kit (Thermo Fisher Scientific, Hemel Hempstead, UK) and a known volume run on a BD Accuri C6+ (BD BioSciences, Wokingham, UK) and analysed with FlowJo software (BD BioSciences, Wokingham, UK). Donor results were then quality-controlled as previously described [13] and the presented data are corrected for background cytokine secretion and expressed as spot-forming units per million CD3+ T cells (sfu/10^6^ CD3+ T cells). A previously established comparison of responses from HCMV-seropositive and HCMV-seronegative donors to HCMV proteins and the positive control [13].

### 2.6. CMV Activation Assay and Spectral Flow Cytometry Acquisition

CMV specific T cells were compared between PBMC and BMMNC samples for all three donors by using a 37-colour cell surface phenotyping and intracellular cytokine staining method. A minimum of 1 × 10^6^ cells, defrosted and overnight rested PBMC and BMMNC from each donor, were resuspended in 250 μL TexMACS in polypropylene FACS tubes. The cells were stimulated with the addition of 180 μL of either TexMACS only, positive control mixture or HCMV ORF mixes, also added were 1/167 dilution CD107a Alexa Fluor 647 antibody and purified co-stimulatory antibodies CD28 (clone CD28-A, Mabtech AB, Nacka Strand, Sweden) and CD49d (clone 9F10, BioLegend, San Diego, CA, USA) at 1/500 dilution. Cells were incubated at 37 °C for 1 h prior to the addition of 1/1000 dilution of brefeldin-A and monensin (BioLegend), cells were mixed and further incubated at 37 °C overnight (maximum 18 h incubation) tilted 5° above horizontal. An unstained sample of PBMC and BMMNC for each donor was also prepared in the same way. Following overnight incubation, the cells were washed in FACS Wash and then stained with a surface antibody mix also containing Brilliant Stain Buffer Plus and LIVE/DEAD fixable Olive Dead cell stain (Thermo Fisher Scientific, Hemel Hempstead, UK) for 1 h at 4 °C. The samples were washed in FACS Wash and then resuspended in Fix&Perm Solution A (Fix) (Nordic-MUbio, Susteren, The Netherlands), mixed and incubated for 30 min at 4 °C. Following a further wash step the supernatant was removed and the cells were permeabilised using Fix&Perm Solution B (Perm) (Nordic-MUbio) and stained with the intracellular antibody mix with Brilliant Stain Buffer Plus added, overnight at 4 °C in the dark. The full details of the spectral panel antibodies are listed in Table S2. The next day cells were washed in excess FACS Wash with 2 mM Sodium Azide (Severn Biotec, Kidderminster, UK) and finally fixed with FluoroFix Buffer. Samples were acquired using a 5-laser configured Sony ID7000 Spectral Cell Analyser (Sony Biotechnology, San Jose, CA, USA) in standardised mode with spectral unmixing and autofluorescence subtraction performed by the Sony system software using a Weighted Least Squares Method (WLSM) algorithm. Spectral references for the unmixing were previously generated using a mixture of bead controls and single colour cell controls and stored in the library for subsequent use. Following acquisition, the unmixed FCS 3.1 files were exported for further downstream analysis.

### 2.7. Analysis of Spectral Flow Data

The spectrally unmixed FCS 3.1 files acquired were imported into FlowJo v10.10.0 and then the parameter names in the FCS file were edited using the FlowJo workspace R plugin Premessa (https://github.com/ParkerICI/premessa, accessed on 19 May 2025) to remove the square brackets present in the Sony ID7000 parameter names. The FCS files were then transformed and scaled and then quality controlled prior to further analysis using the PeacoQC algorithm plugin [41] in FlowJo, with good events exported as a new FCS file. Using the exported good event FCS files further transformation of each parameter was then performed to optimise visualisation of the positive and negative stained populations using FlowJo. The FCS files were further processed applying a conventional gating strategy to exclude forward scatter and then side scatter doublets and dead cells. Live single cells were then gated for CD45 positive cells, followed by exclusion of CD14+ and CD19+ cells and then identification of CD3+ T cells and the CD4+ and CD8+ subsets (workflow and gating shown in Figure S2A). Keywords were added at this stage to identify the donor, the sample type (PBMC or BMMNC) and the stimulation conditions of each sample. Positive and negative expression of the remaining stained protein parameters were determined at this stage using a combination of previous Fluorescence Minus One samples, unstained controls and for the activation and cytokine markers present in the panel the unstimulated and positive control samples.

#### 2.7.1. Resting (Ex Vivo) T Cell Phenotype Workflow for Comparison Between PBMC and BMMNC

To investigate the landscape of differences in marker expression on CD4+ and CD8+ T cells between the ex vivo Bone Marrow and Peripheral Blood compartments, the PBMC and BMMNC unstimulated samples for all three donors were combined into a CD4 file (down sampled to contain 4200 CD4+ T cell events per compartment and donor) and a CD8 file (down sampled to contain 3000 CD8+ T cell events). A dimensionality reduction t-distributed stochastic neighbour embedding (tSNE) visualisation on the four parameters associated with T cell memory subsets (CD45RA, CD27, CD28 and CD57) was performed using the embedded tSNE function in FlowJo [42,43] on the CD4 and CD8 files. FlowSOM clustering algorithms were then applied [44] on the data for different combinations of markers (i) a functional/cytokine marker subset (CD107a, TNFα, IL-2, IL-10 and IFNγ), (ii) an activation/T cell marker subset (CD127 (IL-7Rα), CXCR4 (CD184), CD25 (IL-2Rα), OX-40 (CD134), 4-1BB (CD137), HLA-DR, CD69, CD40L (CD154) and CD39) and (iii) Checkpoint inhibitor Receptor (CIR) panel (TIGIT, CTLA-4 (CD152), 2B4 (CD244), ICOS (CD278), Tim-3, PD-1 (CD279), BTLA (CD272), LAG-3 (CD223), GITR (CD357), KLRG-1 and CD2). The analysis workflow and representative data from the flow cytometry analysis are illustrated in Figure S2B.

#### 2.7.2. Identification of CMV Specific T Cell Responses

Each of the three donors were stimulated with a panel of HCMV proteins which they had responded to in the previous fluorospot assays. The responses to nine different activation associated markers [45,46,47] (4-1BB (CD137), OX-40 (CD134), CD40L (CD154), CD69, CD107a, CD25, PD-1 (CD279), IFNγ and TNFα) following background subtraction of the unstimulated PBMC or BMMNC control were determined for each donor. Representative flow plots results of the Unstimulated control versus US28 stimulation are shown along with heatmaps summarising all the corrected results for the three donors are shown in Figure S3A,B. A positive response to HCMV peptide stimulation by either the PBMC or BMMNC or both for each donor was determined as a minimum of five out of nine responding markers above a threshold of 0.05 following background correction. The summarised results of this determination of a positive CMV protein response are shown in Figure S3C. Using this method the positive control stimulation produced a 9 out of 9 response from all donors in the PBMC samples and BMMNC samples from donors 45793 and 46899 and a 7 out of 9 response from donor 45855 BMMNC sample. In order to compare the phenotype of activated CD4+ and CD8+ T cells to resting cells from each tissue compartment, a Boolean Logic “OR” gate was used to identify activated cells stimulated by the positive control or CMV peptides [48]. The included parameters for the OR gate were 4-1BB+, CD69+, CD40L+, OX-40+, IFNγ+ or IL-10+ responses, and the workflow to generate the final FCS file for high dimensional analysis are summarised in Figure S4A.

#### 2.7.3. CMV IE and US28 Specific T Cell Analysis Workflow

To generate down sampled and concatenated files to analyse changes in T cell phenotype between resting and CMV specific cells the following sequence of concatenation and export of FCS files was performed. A CMV response and Positive control concatenated PBMC and BMMNC file for CD4+ and CD8+ T cells for each donor was generated—down sampled to the lowest number in the CD4+ or CD8+ gate in the 6 or 4 FCS files, respectively. Next the three donor generated files for each condition were concatenated together without down sampling. Finally, to generate a file that contained unstimulated T cells to compare to antigen specific T cells, the OR gate described above was added to each stimulation in the concatenated files and the events in that gate exported as a new FCS file, alongside this a proportionate number of randomly selected unstimulated events from both the PBMC and BMMNC samples within the concatenated file were exported. The exported antigen specific and unstimulated files were then concatenated together to generate four FCS files for high dimensional analysis.

The high dimensional analysis sequence outlined for the resting T cells was also followed on the CMV specific concatenated samples, with an initial tSNE performed based on the four T cell memory parameters (CD45RA, CD27, CD28 and CD57), then additional tSNEs were run that included either the sample type or stimulation condition or both as a parameter. Three FlowSOM clustering algorithms were run based on the same groups of parameters described in the resting analysis—functional, activation/T cell markers and CIRs. The three different FlowSOM maps were then applied to the different stimulation conditions and tissue compartment samples to look for differences between these. The repeated FlowSOM analyses generated heatmaps, frequency distributions and self-organising maps which allows the comparison of bone marrow and peripheral blood derived T cells for the 3 different groups of markers, Figure S4B illustrates the results of this analysis workflow for the CD4 positive control sample. Table S3 summarises the FlowSOM algorithms run for the CMV specific CD4+ and CD8+ T cell analysis.

### 2.8. Statistical Analysis

The processed results from all analyses performed in this study were analysed and visualised using GraphPad Prism version 10 for Windows (GraphPad Software, Boston, MA, USA).

## 3. Results

### 3.1. Analysis of the Cellular CD45+ and CD45− Cell Populations Present in PBMC and Bone Marrow Mononuclear Cells

**Figure 1:**
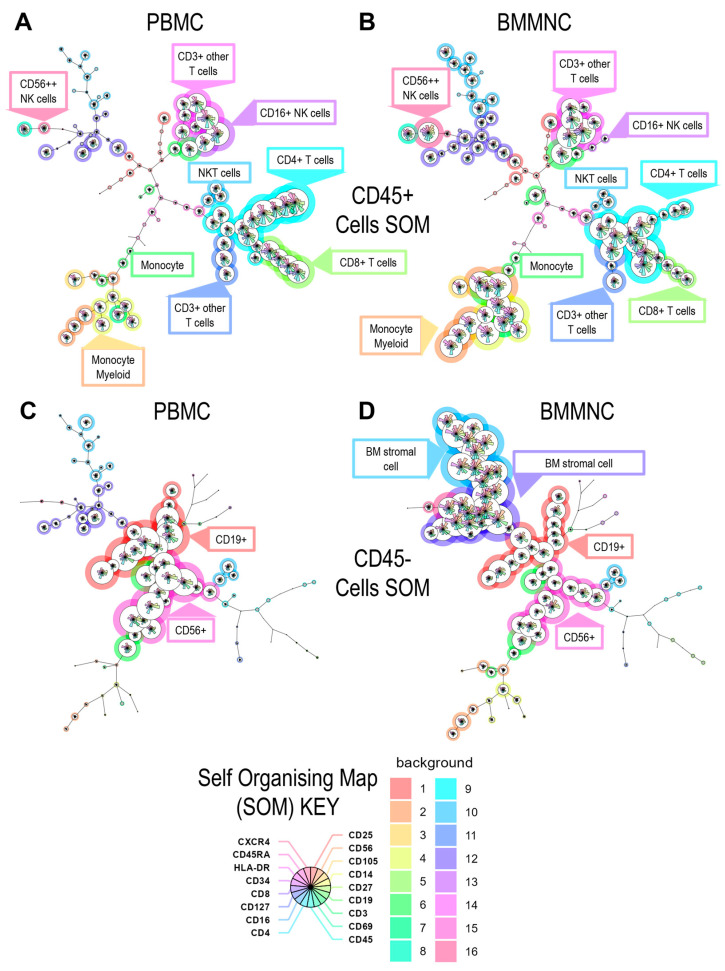
Cellular comparison of PBMC and BMMNC samples. The PBMC and BMMNC samples from the three donors were stained with a panel of antibodies to identify the major lymphocyte and non-lymphocyte cells present in the paired samples by flow cytometry. Following acquisition and analysis the data was quality controlled and then a down-sample of the live cells from each donor and tissue compartment were concatenated together (workflow process is shown in Figure S1). Subsequently, the data was analysed using the FlowSOM algorithm generating the annotated self-organising maps shown for CD45 expressing lymphocytes from the PBMC (**A**), BMMNC (**B**) and CD45 negative PBMC cells (**C**) and BMMNC (**D**). The colours for the 16 metaclusters and the pie charts showing individual marker expression are outlined in the key at the bottom. The major populations that are of interest in each self-organising map are annotated in the figure.

Paired peripheral blood mononuclear cells (PBMCs) and bone marrow mononuclear cells (BMMNCs) isolated at the same time from three different donors were stained with a panel of antibodies and analysed by flow cytometry to assess the lymphocyte composition of CD45+ leukocytes fraction and the stromal/stem cell composition of the CD45− fraction. The analysis revealed that BMMNCs contained CD4+ and CD8+ T cells, composed of various memory subsets, as well as NK cells, CD19+ B cells, and myeloid populations. However, the frequency of these lymphocyte subsets was lower in BMMNCs compared to the matched PBMC samples (Figure 1 and Figure S5).

Examination of the CD45− cell fraction within the BMMNCs showed a markedly higher frequency of CD34+ cell populations relative to peripheral blood, consistent with the expected enrichment of hematopoietic stem and progenitor cells [49,50,51] in bone marrow-derived samples and bone marrow resident stromal cells [52] (Figure 1 and Figure S5).

### 3.2. Analysis of HCMV-Specific T Cell Frequency and Cytokine Responses to Overlapping Peptide Pools Representing HCMV Lytic and Latent Proteins

We previously analysed PBMCs and BMMNCs from the same donor (though not temporally matched) to assess the presence of HCMV-specific CD4+ and CD8+ T cells in the bone marrow and to evaluate differences in the balance of IFN-γ and IL-10-secreting cells between the peripheral blood and bone marrow compartments.

We have now extended and refined this analysis using PBMCs and BMMNCs samples from three additional HCMV-seropositive donors. In addition to measuring IFN-γ and IL-10 we also assessed the anti-viral cytokine TNF-α in response to eight peptide pools derived from immunodominant HCMV proteins expressed during lytic infection (UL83 (pp65), UL144 (with known strain variants included), UL123 (IE1), UL122 (IE2), US3, UL82 (pp71), UL28 and UL55 (gB)), as well as four peptide pools corresponding to proteins also expressed by cells carrying latent HCMV (UL138, LUNA (UL81-82as), US28, UL111A (vIL-10).

**Figure 2:**
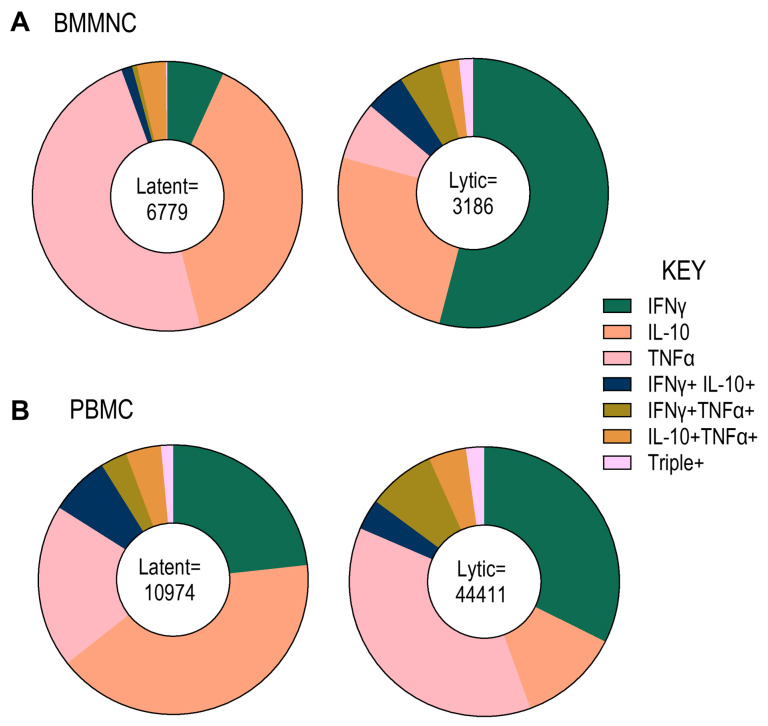
T cells from BMMNC and PBMC produce cytokines in response to HCMV protein stimulation. The production of IFNγ, IL-10 and TNFα by T cells in response to 12 HCMV protein overlapping peptide pools was measured using a triple fluorospot assay. The results were converted into spot forming units per million CD3+ T cells (sfu/CD3+ 10^6^) with background counts for each cytokine subtracted. The average response of the 3 donors are shown as proportions of CD3+ T cells producing single, dual or triple cytokines for the cumulative responses to the latent proteins (US28, UL138, LUNA, UL111a) or the lytic proteins (pp65, UL144, IE1, IE2, US3, pp71, gB, UL28) for the Bone Marrow samples (**A**) and the Peripheral Blood samples (**B**). The figure is the average cumulative sfu/CD3+ 10^6^ cells for each combination indicating the magnitude of the T cell response to HCMV proteins in each tissue compartment.

The Cytokine responses were measured using a triple FluoroSpot assay. The analysis quantified the frequency of cells producing single, dual, or triple cytokines in response to each peptide pool for each donor in both PBMC and BMMNC samples (Supplementary Figure S6). These data were then used to calculate the cumulative average responses to latent and lytic peptide pools, as well as to assess the polyfunctionality of cytokine responses between PBMCs and BMMNCs (Figure 2).

The results revealed that the majority of responding T cells were single cytokine producers, with a smaller proportion producing two cytokines, and only a minor fraction exhibiting triple cytokine production. Notably, a higher frequency of IL-10-producing T cells specific to latent peptide pools was observed in the bone marrow compared to responses against lytic peptides. In contrast, IFN-γ production was the dominant response to lytic peptide stimulation but was substantially reduced following stimulation with latent antigens. Interestingly, TNF-α responses were more prominent following recognition of latent antigens than lytic ones (Figure 2A).

In PBMCs, latent peptide stimulation also induced a higher frequency of IL-10-producing cells compared to lytic peptides, whereas lytic-specific T cells exhibited a greater frequency of IFN-γ and TNF-α production (Figure 2B).

### 3.3. Expression of Anti-Viral Cytokines, Activation Markers, Activating and Inhibitory Checkpoint Receptors by T Cells Derived from Paired Pbmc and Bmmncs Directly Ex Vivo

We aimed to investigate the distribution of checkpoint inhibitory receptors (CIRs) on CD4+ and CD8+ T cell subsets in PBMC and BMMNCs compartments directly ex vivo. Using a panel of antibodies and spectral flow cytometry, for the expression of key antiviral cytokines (IL-2, TNF-α, IFN-γ), the cytotoxicity marker CD107a, and the immunosuppressive cytokine IL-10. In addition, we assessed a range of activation markers as well as co-activating and inhibitory receptor expression.

**Figure 3:**
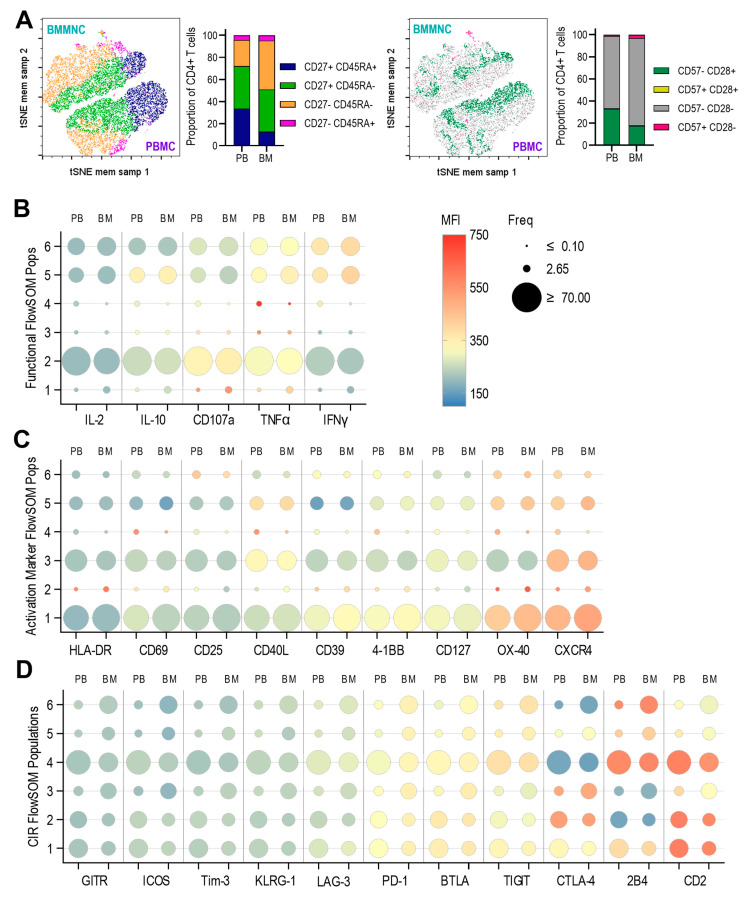
Comparison of resting CD4+ T cells from PBMC and BMMNC samples. The paired samples from the three donors were analysed by spectral flow cytometry using a 37-parameter panel. The resulting data was analysed as outlined in the methods and Figure S2. The concatenated data file was analysed using dimensionality reduction (tSNE) and then clustering analysis (FlowSOM). The proportions of CD27 and CD45RA defined memory subsets and CD57 and CD28 defined differentiation phenotypes are shown as bar graphs and tSNE with populations overlaid (**A**). Summarised results from the three separate clustering analyses are shown for the functional (IL-2, IL-10, CD107a, TNFα, IFNγ) parameters (**B**), activation and T cell markers (HLA-DR, CD69, CD25, CD40L, CD39, 4-1BB, CD127, OX-40, CXCR4) parameters (**C**) and Checkpoint Inhibitor Receptor (CIR) (GITR, ICOS, Tim-3, KLRG-1, LAG-3, PD-1, BTLA, TIGIT, CTLA-4, 2B4, CD2) parameters (**D**). The size of the bubble plots indicates the frequency of the distribution of the 6 clusters between the PBMC and BMMNC samples and the heatmap colour the intensity of each marker’s expression as generated by the FlowSOM algorithm.

Analysis of CD4+ T cell subsets in PBMCs and BMMNC from all three paired donors—defined by surface expression of CD45RA, CD27, CD28, and CD57—revealed that PBMCs contained the expected memory subsets (naïve T cells, central memory (Tcm), effector memory (Tem), and effector memory cells re-expressing CD45RA (Temra). Notably, all these subsets were also present in BMMNC samples (Figure 3A). However, the relative proportions of the subsets differed: BMMNC contained substantially fewer CD45RA+CD27+ cells and a higher proportion of CD45RA−CD27− cells, which were predominantly CD28−CD57− (Figure 3A).

As expected for CD4+ T cells isolated directly ex vivo, most cells did not express—or expressed only very low levels of—intracellular cytokines and CD107a. A small population in both PBMCs and BMMNC did exhibited higher levels of intracellular TNF-α, IFN-γ, and CD107a (presumably activated cells that had recently encountered antigen). However, there were no significant differences in this pattern between PBMC- and BMMNC-derived CD4+ T cells (Figure 3B) and Self Organising Map (SOM) showing the relationships between these populations (Figure S7A).

We also assessed the expression of activation markers (HLA-DR, CD69, CD25), in agreement with the intracellular cytokine data, these were not expressed by the majority of cells, again, higher expression observed in some minor subpopulations in the bone marrow compartment. Most CD4+ T cells expressed some level of the activation marker OX-40 and the bone marrow stroma homing chemokine receptor CXCR4, with increased expression in the same subpopulations (e.g., FlowSOM population 5) that also expressed higher levels of CD40L. Higher levels of CXCR4 expression and increased frequency of the population in the bone marrow for FlowSOM population 1 was also associated with increased expression of CD39 and 4-1BB with concurrent decreased expression of CD127 (Figure 3C). Together with Self Organsing Map (SOM) comparing the PBMC and BMMNC populations from the three donors (Figure S7B) shows that the Bone Marrow is enriched for resting CD4+ T cells that exhibit bone marrow homing and increased expression of activation markers and marker patterns suggestive of regulatory T cell function.

Finally, we examined the direct ex vivo expression of a panel of CIRs. Most resting CD4+ T cells either lacked or expressed only low levels of the CIRs analysed (Figure 3D and Self Organising Map (SOM) showing the relationships between these populations (Figure S7C). However, CTLA-4, 2B4, and CD2 showed differential expression, with certain CD4+ T cell populations exhibiting markedly higher levels. Notably, CTLA-4 and 2B4 expression patterns were often reciprocal: CTLA-4 was low in populations 6 and 4 and high in populations 2 and 3, whereas 2B4 was highly expressed in populations 6 and 4 and low in populations 2 and 3. CD2 was highly expressed in both PBMC and BMMNC samples in populations 1, 2, and 4. Differences in the frequency of CIR-expressing cells between PBMC and BM were most pronounced for CTLA-4, 2B4, and CD2.

**Figure 4:**
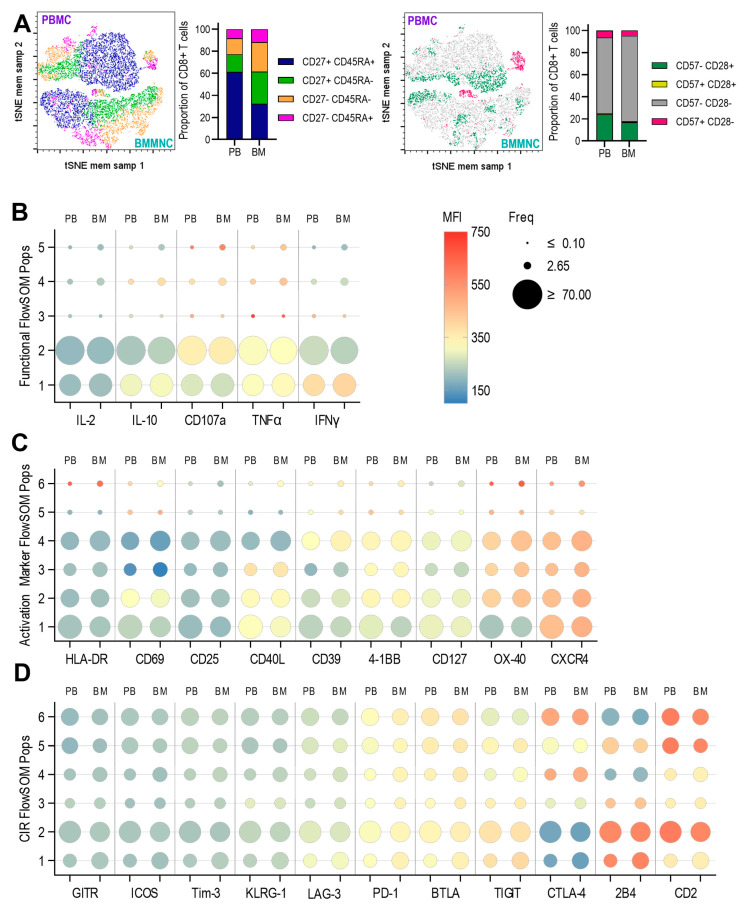
Comparison of resting CD8+ T cells from PBMC and BMMNC samples. The paired samples from the three donors were analysed by spectral flow cytometry using a 37-parameter panel. The data was analysed as previously outlined (Figure S2). Next the concatenated data file was dimensionally reduced (tSNE) and then repeated clustering analyses (FlowSOM) performed. The proportions of CD27 and CD45RA defined memory subsets and CD57 and CD28 defined differentiation phenotypes are shown as bar graphs and tSNE with populations overlaid (**A**). Summarised results from the three separate clustering analyses are shown for the functional (IL-2, IL-10, CD107a, TNFα, IFNγ) parameters (**B**), activation and T cell markers (HLA-DR, CD69, CD25, CD40L, CD39, 4-1BB, CD127, OX-40, CXCR4) parameters (**C**) and Checkpoint Inhibitor Receptor (CIR) (GITR, ICOS, Tim-3, KLRG-1, LAG-3, PD-1, BTLA, TIGIT, CTLA-4, 2B4, CD2) parameters (**D**). The size of the bubble plots indicates the frequency of the distribution of the 5 or 6 clusters between the PBMC and BMMNC samples and the heatmap colour the intensity of each marker’s expression as generated by the FlowSOM algorithm.

Similarly to the results observed for CD4+ T cells, the CD8+CD45RA+CD27+ T cell subset constituted the largest proportion in PBMCs but was nearly halved in frequency in the BMMNC. While the proportion of CD45RA+CD27− (Temra) cells was comparable between PBMC and BMMNC, the BMMNC contained a higher frequency of CD45RA-CD27+/− cells. Nevertheless, it was also notable that all CD8+ T cell subsets identified in PBMCs were also present in the BMMNC (Figure 4A).

CD8+ T cells expressed little to no intracellular antiviral cytokines or CD107a directly ex vivo. As with CD4+ T cells, minor populations in both PBMC and BMMNC expressed high levels of TNF-α, IFN-γ, and CD107a. However, there were no significant differences in these expression patterns between PBMC- and BM-derived CD8+ T cells (Figure 4B and Self Organising Map (SOM) showing the relationships between these populations (Figure S8A)). This trend was also observed for activation markers and for OX40 and CXCR4 expression. Similarly to the pattern of expression observed in resting CD4+ T cells, in FlowSOM population 4 of the resting CD8+ T cell analysis there is increased frequency and expression of CD39, 4-1BB, OX-40 and CXCR4 in the bone marrow samples with decreased CD127 expression. Confirming, the observation that as well as a slightly increased activation phenotype the bone marrow is also enriched for memory T cells with regulatory phenotypes.

Analysis of CIR expression revealed no or very low resting expression of GITR, ICOS, Tim-3, KLRG1, LAG-3, PD-1, BTLA, and TIGIT across all populations. However, as observed in CD4+ T cells, CTLA-4 and 2B4 exhibited a reciprocal expression pattern, and three populations expressed high levels of CD2, while three others showed substantially lower expression. Overall, these distribution patterns were consistent between PBMC and BMMNC samples (Figure 4D and Self Organising Map (SOM) showing the relationships between these populations Figure S8C).

### 3.4. Expression of Anti-Viral Cytokines, Activation Markers, Activating and Inhibitory Checkpoint Receptors in Pbmc and Bmmncs Following Hcmv Antigen Stimulation

As described in Figure 2, comparison of antiviral TNF-α and IFN-γ responses versus IL-10 responses in PBMC and BMMNC compartments revealed a more IL-10–skewed response among T cells specific for latent HCMV antigens in the BMMNC compartment. Notably, the bone marrow—and particularly CD34+ stem cells—is a recognised reservoir for cells harbouring latent HCMV. Given this, it is likely that T cells within the bone marrow are exposed to antigen presentation of latency-associated proteins, but not to proteins associated with the lytic phase of HCMV infection.

We therefore aimed to characterise the CD4+ and CD8+ T cell populations specific for HCMV latent and lytic antigens across these two compartments, with a particular focus on the expression of activation markers, intracellular cytokine production, and changes in checkpoint inhibitory receptor (CIR) expression.

We selected immediate early (IE) antigen-specific responses as a representative example of a classical lytic HCMV protein, and US28-specific responses as a well-characterised latent-phase HCMV protein critical for the maintenance of latency. Additionally, all three donors had a positive response to stimulation by these two proteins (summarised Figure S3C). Responding CD4+ and CD8+ T cells were identified based on activation marker expression post-stimulation and were further characterised for antiviral cytokine production, CD107a expression (as a marker of cytotoxicity), and IL-10 expression. In addition, changes in CIR expression were assessed relative to unstimulated control T cell populations from PBMC and BMMNC, respectively.

**Figure 5:**
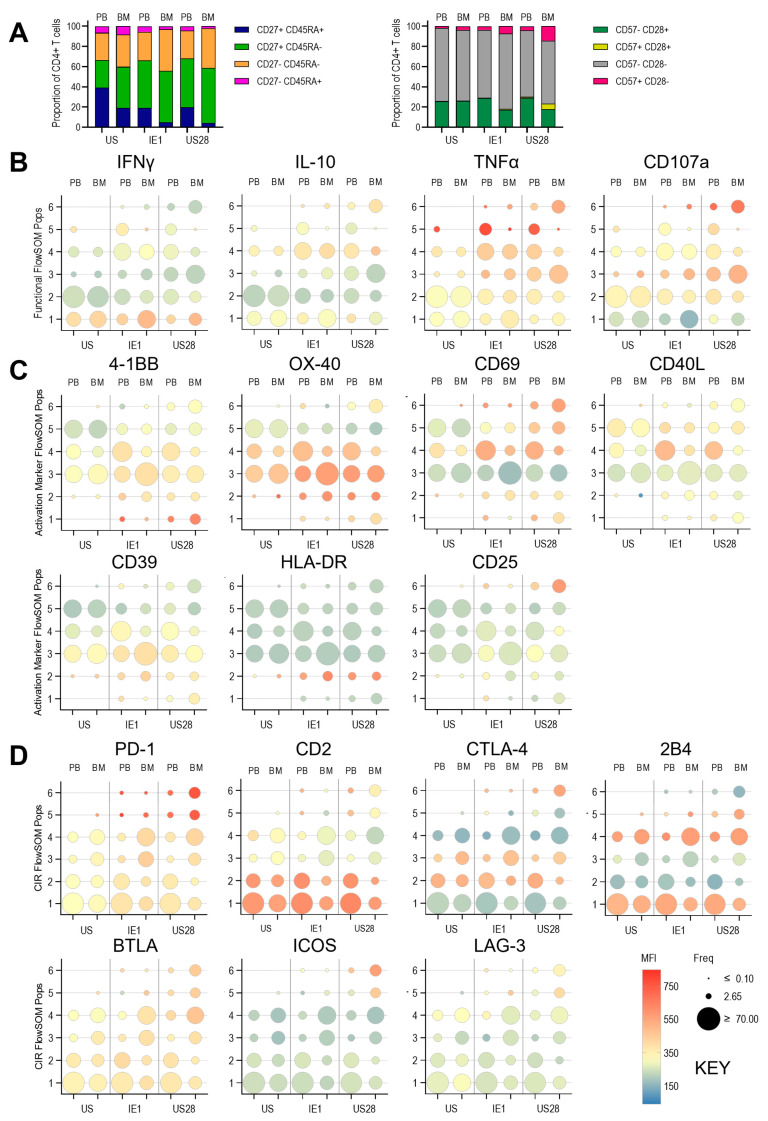
Characterisation of the Latent (US28) and Lytic (IE1) specific CD4+ T cell responses in PBMC and BMMNC. Mononuclear cell samples were stimulated with HCMV peptides overnight and then stained with a 37-parameter panel to identify activated and cytokine secreting cells and their memory and inhibitory receptor phenotype. HCMV specific T cells were identified and concatenated into one file as detailed in the methods and Figures S3 and S4. The memory and differentiation phenotype for the unstimulated and the IE1 and US28 specific CD4+ T cells are shown (**A**). Summarised are individual parameter bubble plots showing the frequency of distribution of the 6 FlowSOM clusters for the PBMC (PB) and BMMNC (BM) samples from all three donors for each stimulation. The bubble plots are coloured according to the intensity of expression as illustrated in the heatmap bar in the key to the graphs. Shown are the results of 4 Functional FlowSOM parameters (**B**), 7 Activation clustering parameters (**C**) and 7 CIR FlowSOM parameters (**D**).

The IE- and US28-specific CD4+ T cells were predominantly CD45RA−CD27+/− and largely CD57−CD28−, with comparable distributions between PBMC and BMMNC compartments. IE1 stimulation induced higher levels of IFN-γ than US28, with a greater frequency of responding cells observed in the BMMNC, which was consistent with the fluorospot assays performed on the same samples. Both IE and US28 stimulation induced IL-10 expression, with US28 eliciting responses in a greater number of populations and at higher levels. TNF-α induction was highest in specific CD4+ T cell populations. Notably, TNF-α–high IE- and US28-specific T cells in population 5 were more frequent in PBMCs, whereas population 6, which was predominantly US28-specific and enriched in the BMMNC, also showed elevated expression of the cytotoxicity marker CD107a (Figure 5B). The distinct differences in the clustered populations between the two tissue compartments and peptide stimulation are clearly seen in the self-organised maps (SOMs) for this analysis (Figure S9B).

IE- and US28-specific cells were identified by the expression of a range of activation markers (Supplementary Figure S4A). T cells activated by either peptide pool exhibited distinct activation marker profiles, which varied between PBMC and BMMNC compartments and between the HCMV lytic and latent peptide pools. Most notably, CD69, CD25, and HLA-DR showed differential expression patterns (Figure 5C). CD39 was expressed by population 3 in this clustering analysis to similar levels in PBMC and BMMNC in unstimulated CD4+ T cells, post stimulation with either peptide pool there is an increase in expression in population 3 as well as the a frequency increase in populations 2 and 3 (Figure 5C) and Self Organising Map (SOM) showing the relationships between these populations (Figure S9C).

**Figure 6:**
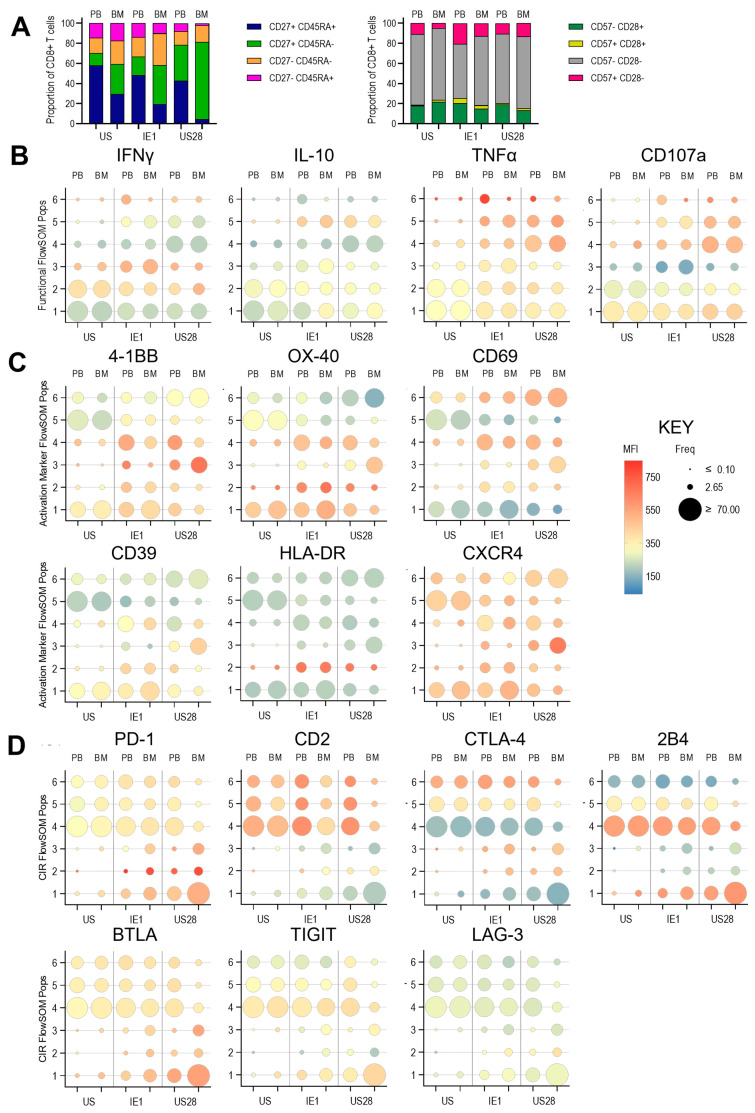
Characterisation of the US28 and IE1 specific CD8+ T cell responses in PBMC and BMMNC samples. Mononuclear cell samples from all three donors were stimulated with HCMV peptides overnight and then stained with a 37-parameter panel to identify activated and cytokine secreting cells and their memory and inhibitory receptor phenotype. HCMV specific T cells were identified and concatenated into one file as previously described (Figures S3 and S4). The memory and differentiation phenotype for the unstimulated and the IE1 and US28 specific CD8+ T cells are shown (**A**). Summarised are individual parameter bubble plots showing the frequency of distribution of the 6 FlowSOM clusters for the PBMC (PB) and BMMNC (BM) samples from all three donors for each stimulation. The bubble plots are coloured according to the intensity of expression as illustrated in the heatmap bar in the key to the graphs. Shown are the results of 4 Functional FlowSOM parameters (**B**), 6 Activation clustering parameters (**C**) and 7 CIR FlowSOM parameters (**D**).

Strong PD-1 induction was observed in BMMNC-derived CD4+ T cells following both IE and US28 stimulation, with US28 eliciting particularly strong responses in populations 5 and 6. Additionally, BMMNC populations expressed high levels of CTLA-4, BTLA, ICOS, and LAG-3, especially following US28 stimulation, compared to unstimulated controls (Figure 5D). Additionally, US28 specific T cells in the bone marrow clustered in populations 5 and 6 also expressed higher levels of BTLA and ICOS—suggesting that overall, the bone marrow resident US28 specific CD4+T cells have a more inhibitory phenotype. In contrast to the CD4+ T cell data, peptide-specific CD8+ T cell responses differed in both subset distribution and compartmental localisation. IE-specific CD8+ T cells in PBMCs showed a distribution similar to that of unstimulated controls, whereas in the BMMNC, responding cells were enriched in CD45RA+CD27+/− subsets. US28-specific CD8+ T cells in PBMCs showed a shift toward CD45RA−CD27+ cells, while in the BMMNC, this subset was the dominant responding population (Figure 6A).

IFN-γ production was observed following both IE and US28 stimulation, particularly in populations 3 and 6. IL-10–producing cells were enriched in population 5, with increased expression intensity across populations 1, 2, and 3 compared to unstimulated controls. TNF-α and CD107a expression were notably elevated in populations 4, 5, and 6, with similar distribution patterns observed between PBMC and BMMNC compartments (Figure 6B). In particular population 4 was increased in frequency (also seen in self-organising map Figure S10B) following US28 stimulation and the bone marrow resident CD8+ T cells have a strong TNFα and CD107a expressing anti-viral phenotype.

Peptide stimulation also induced distinct activation marker profiles across CD8+ T cell populations, particularly for 4-1BB, OX40, CD69, and HLA-DR (Figure 6C). Similarly to the stimulation of CD4+ T cells, CD39 expression is increased in some responding populations in both PBMC and BMMNC however there is a notable increase in both expression and frequency in some specific populations in the BMMNC (polulation1 following IE and population 3 following US28 stimulation (Figure 4C) the SOM also showing the relationships between these populations (Figure S10C).

Induction of the CIRs PD-1, BTLA, TIGIT, and LAG-3 was most prominent in populations 1, 2, and 3, with higher frequencies in BMMNC-derived cells, particularly following US28 stimulation. These populations also exhibited notably lower CD2 expression. CTLA-4 was induced in populations 2 and 3, and 2B4 was upregulated in population 1, with both markers showing higher expression in BMMNC compared to PBMCs (Figure 6D). The distinct distribution of the frequency of IE1 and US28 specific CD8+ T cells between the different clustered populations seen between the PBMC and BMMNC compartments is particularly clearly seen in the self-organising map comparison (Figure S10D).

The previous fluorospot screening data suggested an enrichment of IL-10–secreting T cells residing in the bone marrow. Therefore, we further investigated the expression of checkpoint inhibitory receptors (CIRs) and immunomodulatory markers on CMV-specific IL-10–secreting CD4+ and CD8+ T cells. Compared to IFNγ-secreting CMV-specific T cells, IL-10–producing CD4+ T cells responding to IE1 and US28 stimulation exhibited increased expression of BTLA, CD25, CD39, CTLA-4, and PD-1. Notably, ICOS, LAG-3, and Tim-3 expression was also elevated on IL-10+ US28-specific bone marrow CD4+ T cells (Figure S11A).

A similar phenotype was observed in CD8+ CMV-specific IL-10+ T cells. PD-1 expression was markedly upregulated in both IE1- and US28-responsive cells, while CD39 expression was particularly elevated in IE1-specific IL-10–secreting CD8+ T cells. US28-specific IL-10+ CD8+ T cells in the bone marrow also showed increased expression of GITR, ICOS, LAG-3, and CD25 (Figure S11B).

Importantly, IL-10– and IFNγ–secreting T cells represented distinct cellular subsets. Interestingly, TNFα secretion was predominantly observed within the IL-10–producing subset. Collectively, these findings suggest that CMV-specific IL-10–secreting T cells in the bone marrow exhibit elevated expression of multiple inhibitory receptors and immunomodulatory markers compared to their non–IL-10–producing counterparts.

## 4. Discussion

In this manuscript we describe a comparative analysis of peripheral blood and bone marrow mononuclear cells (PBMCs and BMMNCs) from three HCMV seropositive participants. The analysis revealed distinct immunological landscapes, with BMMNCs showing reduced frequencies of major lymphocyte subsets but enrichment of CD34+ hematopoietic progenitors. Functional assays highlighted compartmentalised T cell responses to HCMV antigens, with BMMNCs exhibiting a predominance of IL-10 and TNF-α responses to latent antigens and a more immunoregulatory phenotype, characterised by distinct cytokine profiles, memory subset distributions, and checkpoint inhibitory receptor expression.

Many previous studies investigating CMV-specific T cell responses in the bone marrow have primarily identified responses to the immunodominant lytically expressed proteins pp65 and IE [21,35,36,37,38]. In earlier work, we analysed T cell responses in a single donor to a range of HCMV proteins associated with both latent and lytic infection [11,34]. This preliminary analysis revealed that the bone marrow harbours T cell responses to a broad array of HCMV proteins, including IL-10–secreting T cells specific for antigens that are rarely detected in peripheral blood.

In the present study, we expanded our analysis to include three cytokines—IFNγ, IL-10, and TNFα—using donor-matched PBMC and BMMNC samples collected simultaneously. This approach enabled a more accurate comparison of HCMV-specific T cell responses across tissue compartments. When comparing responses across all three donors to latency-associated and lytically expressed proteins, we observed distinct cytokine-secreting phenotypes. Notably, CMV-specific T cells in the BMMNC compartment were predominantly single-cytokine producers, whereas PBMC-derived T cells exhibited a higher proportion of dual- and triple-cytokine–secreting cells. This contrasts with previous findings in which bone marrow–resident CMV pp65-specific T cells were largely polyfunctional [21].

The cytokine response profile in bone marrow T cells also differed markedly between latent and lytic protein groups. IL-10 and TNFα dominated responses to latent antigens, while IFNγ was the primary cytokine induced by lytic proteins. Interestingly, a quarter of bone marrow–resident T cells responding to lytic proteins produced IL-10 alone—twice the frequency observed in PBMCs—supporting our earlier findings that IL-10–secreting CMV-specific T cells are enriched in the bone marrow [11,34]. PBMC responses to latent protein stimulation also included IL-10–producing cells, consistent with our previous work showing that IL-10 secretion is a common feature of both CD4+ and CD8+ T cell responses to latency-associated proteins [11,13].

The increased proportion of TNFα-secreting T cells in the bone marrow in response to latent protein stimulation is particularly noteworthy. Although there was considerable inter-donor variability—ranging from 20% to 70% of bone marrow–resident T cells producing TNFα—all three donors exhibited higher TNFα responses to latent proteins compared to lytic proteins. TNFα is generally regarded as a pro-inflammatory cytokine and has been shown to contribute to the formation of an inflammatory microenvironment in the bone marrow [53]. It has also been implicated in the promotion of regulatory T cell function in several studies [54]. Moreover, recent findings suggest that TNFα plays a critical role in regulating the plasma cell niche within the bone marrow [55]. These observations raise the intriguing possibility that CMV may modulate the bone marrow immune environment through the induction of TNFα.

While fluorospot assays are highly sensitive for detecting cytokine-secreting T cells, they do not provide phenotypic information about the cytokine-producing populations. To fully characterise bone marrow–resident CMV-specific CD4+ and CD8+ T cells, including cytokine production, cytotoxic markers, and detailed phenotyping, we performed a spectral flow cytometry–based CMV activation assay. This 37-parameter panel also enabled a comprehensive comparison of the resting T cell phenotypes in bone marrow versus peripheral blood.

Our initial 18-parameter flow cytometry panel, applied to samples from three donors, revealed that T cells are proportionally less abundant in bone marrow aspirates compared to peripheral blood. Additionally, the non-lymphocyte component of the bone marrow was markedly different from that of PBMCs, with likely presence of bone marrow mesenchymal stromal cells [52], identified in our analysis as CD45−CD34+CD56+ cells. This difference also implies that the composition of antigen-presenting cells (APCs) capable of presenting CMV peptides differs substantially between BMMNC and PBMC samples, reflecting the distinct immunological microenvironments of these compartments in vivo.

Analysis of resting T cell populations in the bone marrow revealed subtle but consistent differences compared to PBMCs. These included an enrichment of memory T cells, a corresponding reduction in naïve T cells, and increased background cytokine production and expression of certain activation markers [21,29]. Although bone marrow T cells often exhibit a more activated phenotype, previous studies using proliferation markers have confirmed that these cells are functionally resting [21,26,29,33]. However, these resting memory T cells retain the ability to rapidly respond to peripheral antigenic re-challenge in the absence of persistent antigen expression in the bone marrow [22,30,38].

Our comparative analysis of bone marrow–resident CMV-specific T cells focused on responses to the lytically expressed protein IE1 and the latency-associated protein US28. This focus was informed by earlier cytokine profiling, which suggested potential differences in the phenotypes of T cells responding to these two antigens. The spectral flow cytometry results supported this hypothesis, revealing clear phenotypic differences between IE1- and US28-specific CD4+ and CD8+ T cells in both BMMNC and PBMC samples. Notably, clustering analyses showed striking differences in the phenotypic profiles of US28-specific T cells between PBMC and BMMNC compartments.

These differences in bone marrow–resident memory T cell phenotypes between US28 and IE1 are likely attributable to the underlying biology of CMV infection. The bone marrow, particularly CD34+ progenitor cells, is a well-established site of HCMV latency [18,19]. A defining feature of latent infection is the repression of immediate early and early viral gene expression, thereby preventing viral replication and reactivation [14]. However, we and others have shown that a limited set of viral genes are expressed during latency to maintain the latent state [9,56] and overall full lytic gene expression is repressed in these latent infected cells. Consequently, it is unlikely that lytic antigens such as IE1 are normally present in this environment, although memory T cell responses to systemic pathogens can persist in the bone marrow even in the absence of antigen [23].

IE1-specific T cells are undoubtedly present in the bone marrow and would be capable of responding if exposed to antigen. We have previously demonstrated that IE protein expression can be induced when latency-maintaining mechanisms are disrupted, for example, through the use of histone deacetylase inhibitors (HDACi) [57], bromodomain and extraterminal domain inhibitors (I-BET) [58], or signal-blocking nanobodies targeting US28 [59]. Such interventions enable IE-specific T cells to recognise antigen and eliminate latently infected cells.

In contrast, US28, a virally encoded G protein-coupled receptor (GPCR) homologous to CX3CR1 [60] can be expressed on the surface of latently infected cells and is essential for the maintenance of latent infection [5,6,61,62]. It is likely that US28 is expressed within the bone marrow microenvironment. Due to the repression of gene expression during latency [14], viral genes involved in immune evasion through MHC class I and II downregulation, principally US2–US11, are not expressed [63,64]. As a result, the virus likely employs alternative mechanisms to evade immune detection during latency, particularly within immune-privileged sites such as the bone marrow.

We have previously identified latency-associated protein–specific CD4+ and CD8+ T cells with cytotoxic and antiviral properties. However, these T cells also secrete the immunosuppressive cytokine IL-10 [10,11,12,13]. In addition to inducing host IL-10 production, the virus encodes a viral IL-10 homolog, UL111A, which is expressed during latent infection [3] and is capable of downregulating MHC class II expression [65]. Furthermore, latently infected cells contribute to the creation of an immunosuppressive microenvironment [17] by modulating the secretion of host proteins from infected CD34+ progenitor cells [15] and CD14+ monocytes [16], thereby impairing antiviral T cell effector functions.

The results of this study suggest an additional mechanism by which HCMV may modulate the immune response in the bone marrow: by increasing the expression of checkpoint inhibitory receptors on CMV-specific T cells. Notably, our clustering analysis revealed elevated expression of CD39, PD-1, BTLA, CTLA-4, ICOS, and LAG-3 on CD4+ T cells, and increased expression of PD-1, CD39, BTLA, TIGIT, LAG-3, and ICOS on CD8+ T cell populations enriched for US28 specificity in the bone marrow. While increased expression of inhibitory molecules has previously been described on tissue-resident T cells [66], an in-depth characterisation of the inhibitory receptor profiles of CMV-specific bone marrow–resident T cells has not been conducted. Many of these identified molecules have been previously associated with CMV-specific T cell responses.

We and others have reported that PD-1 expression on T cells is associated with an IL-10–secreting [11] and often cytotoxic phenotype [67]. In murine CMV (MCMV) infection models, CD8+ T cells in the salivary glands express multiple exhaustion-associated markers, including PD-1 [68]. In a humanised mouse model, PD-1 upregulation on CMV-specific effector CD4+ T cells in the spleen emerged as a robust marker of HCMV reactivation [69]. In clinical settings, increased PD-1 expression has been observed in viremic versus non-viremic kidney transplant patients [70], and in stem cell transplant recipients, elevated PD-1 levels were associated with viremia and acute graft-versus-host disease [71]. CTLA-4 is typically expressed by activated T cells and transmits inhibitory signals to regulate antigen-specific proliferation [72]. During primary CMV infection, CTLA-4 is upregulated, suggesting a role in the establishment, expansion, and function of CMV-specific T cells [70]. However, blocking CTLA-4 in CMV-specific T cells did not fully reverse the suppressive effects of CMV-specific regulatory T cells [73]. Other studies have shown that combined blockade of CTLA-4 and PD-1 can enhance T cell responses, particularly in the context of chronic CMV infection [74].

Other CD28 superfamily molecules upregulated in US28-specific bone marrow T cells is B and T lymphocyte attenuator (BTLA) [75] and Inducible Costimulator (ICOS) [76], both of which are rapidly induced following T cell activation [76,77]. During primary CMV infection, BTLA expression is upregulated on CMV-specific CD8+ T cells during their transition from naïve to effector states and subsequently downregulated in memory populations [78]. Elevated BTLA expression has also been observed in donor-positive/recipient-negative transplant patients and in individuals experiencing CMV reactivation [79]. The inhibitory role of BTLA in CMV infection was further supported by a blockade study in kidney transplant patients, where inhibition of BTLA led to increased production of antiviral cytokines [80]. BTLA binds to the herpesvirus entry mediator (HVEM) [77], and CMV encodes an HVEM orthologue, UL144, which specifically interacts with BTLA to inhibit T cell activation, thereby contributing to viral persistence [81]. Expression of ICOS is upregulated on CMV-specific CD8+ TEMRA cells following stimulation with peptide-pulsed monocytes [82]. Along with PD-1, ICOS expression is a hallmark of activated T follicular helper (Tfh) cells, which emerge early during primary CMV infection and contribute to enhanced antibody responses [83]. The importance of ICOS in modulating the immune response to CMV is further underscored by the observation that both HCMV and MCMV encode proteins that downregulate its ligand, ICOSL, resulting in increased viral loads in the salivary glands of infected mice [84]. Additionally, ICOS has been shown to be required for IL-10 production by T cells in the salivary gland in a separate MCMV model [85].

On bone marrow–resident CD8+ T cells specific for US28, we also observed increased expression of TIGIT (T cell immunoglobulin and ITIM domain), a member of the nectin and nectin-like family of proteins. TIGIT is an inhibitory receptor structurally related to the activating receptor DNAM-1 (CD226) and the inhibitory receptor CD96 [86]. TIGIT, DNAM-1, and CD96 share ligands expressed on antigen-presenting cells, including CD155 (PVR), with TIGIT showing preferential binding to PVR [86]. Increased TIGIT expression has been observed in end-stage lung transplant patients following pp65 and IE1 stimulation [87], and in kidney transplant recipients, a higher frequency of TIGIT expressing effector memory CD4+ T cells has been associated with post-transplant CMV infection [88]. Overall, TIGIT appears to diminish T cell effector function, either through the induction of IL-10 production [89] or via other mechanisms, particularly in the context of persistent viral infections [90].

Lymphocyte Activation Gene 3 (LAG-3) is a structural homolog of CD4 and employs multiple mechanisms to inhibit T cell function [91]. It interacts with MHC class II and several other ligands, including galectin-3 [92], and binds MHC class II with higher affinity than CD4 [93]. LAG-3 has been consistently associated with T cell exhaustion in chronic infections, particularly in the murine LCMV model, where LAG-3 blockade improves T cell responses and reduces viral load [94]. In HIV patients, increased LAG-3 expression on both CD4+ and CD8+ T cells correlates with disease progression [95]. In CMV infection, LAG-3 expression is elevated on tetramer-positive CD8+ T cells during primary infection [96], and in patients with end-stage lung disease awaiting transplant, LAG-3 is induced on all CMV-specific T cells following pp65 and IE peptide stimulation [87]. Notably, in CMV-specific T cells used for adoptive cell therapy, LAG-3 expression was upregulated on pp65-reactive CD8+ T cells, and this expression was further enhanced when Tim-3 was blocked [97].

In our previous investigations of CMV-specific IL-10–secreting CD8+ T cells, we identified increased expression of CD39—either alone or in combination with PD-1—as a defining feature of these subsets [11]. CD39 is an ectoenzyme which catalyses the hydrolysis of extracellular ATP, ultimately producing immunosuppressive extracellular adenosine [98]. In CMV infection, high CD39 expression has been associated with inducible regulatory T cell (iTreg) phenotypes and is particularly elevated in patients with recurrent CMV disease [99]. CD39 is also highly expressed on tissue-resident memory T (Trm) cells in the brain, lung, and skin, and is especially prominent on EBV-specific CD8+ T cells isolated from melanoma tumours [100]. In murine CMV models, tissue-resident CD8+ T cells express high levels of PD-1 and CD39. Interestingly, CD39 expression on salivary gland CD8+ T cells is maintained independently of ongoing viral replication or antigen presence [68], which may explain the increased CD39 expression observed on IE-reactive T cells in the bone marrow in this study.

The findings presented in this study, along with our previous work on functional anti-CMV immune responses in the elderly [101], highlight the critical role of the local microenvironment in shaping the effectiveness of CMV immunity. The bone marrow aspirate samples analysed contain a markedly different composition of non-lymphocyte cells compared to peripheral blood, which is more commonly studied. It is plausible that the distinct repertoire of checkpoint ligands expressed by bone marrow antigen-presenting cells also contributes to the differences observed in this study. We have previously shown that the expression of ligands such as PD-L1, B7-H3, and HLA-E is elevated on fibroblasts from older donors [101], and it is likely that the presence of latent or reactivating virus in the bone marrow influences the expression of these ligands. A comprehensive understanding of the differences between T cells from various tissue compartments will require detailed profiling of the ligands expressed by local antigen-presenting cells.

Future single-cell RNA sequencing studies would allow us to fully elucidate the differences in the landscape of antigen-presenting cells and stromal cells in the bone marrow compared to peripheral blood. Such studies would also enable the characterisation of TCR usage by CMV-specific T cells in each compartment, as well as the assessment of transcription factor expression and epigenetic signature differences in bone marrow-resident T cells [33,102]. These investigations will contribute to a more comprehensive understanding of the CMV memory response in the bone marrow and help identify key parameters for use in larger human studies, where sample availability may be limited.

In this study, we used overlapping peptide pools of CMV proteins to identify CMV-specific T cells. However, to more accurately reflect in vivo conditions, future studies should employ in vitro CMV infections of autologous antigen-presenting cells derived from bone marrow and other relevant tissues. This approach would allow us to assess how CMV infection alters the expression of surface ligands and how these changes influence the upregulation of corresponding checkpoint receptors on CMV-specific T cells.

The overall inhibitory phenotype of bone marrow–resident CMV-specific T cells contributes to our current understanding of the aetiology of CMV infection and the mechanisms underlying the maintenance of latency. Importantly, the inhibitory molecules identified in this study are already of significant interest in the field of cancer immunotherapy, with several checkpoint inhibitors currently in clinical use. Our findings identify a range of potential therapeutic targets that could be leveraged to expand treatment options for severe CMV disease.

## Figures and Tables

**Table 1:** Paired PBMC and BMMNC Donor Characteristics.

Donor ID	Age	Sex	HLA Class I
45739	25	Male	A01 A32; B08 B41; C07 C17
45855	31	Male	A03 A30; B35 B81; C04 C08
46899	24	Male	A31 A36; B15 B53; C04 C08

## Data Availability

The data presented in this study are available on request from the corresponding authors.

## Supplementary Materials

The following supporting information can be downloaded at: https://www.mdpi.com/article/10.3390/pathogens14080722/s1. It includes Supplementary Table S1: Major lymphocyte subsets’ antibody panel details; Table S2: 37 Spectral panel antibody clone and manufacturer details, Table S3: High Dimensional Flowcytometry analysis; FlowSOM algorithm runs in FlowJo v10.10. Supplementary Figure S1: Major Lymphocyte subsets workflow and representative flow cytometry plots, Figure S2: Resting cell analysis spectral workflow and representative gating, Figure S3: Determining HCMV-specific T cell responses following spectral flow cytometry, Figure S4: Workflow for selecting antigen-specific T cells and subsequent analysis using the CD4+ positive control as a representative example, Figure S5: Frequency and marker expression intensity of the CD45 positive and CD45 negative analysis of the major cellular populations’ clustering analysis, Figure S6: Magnitude and cytokine secreting composition of PBMC and BMMNC from the three donors for the individual HCMV, Latent, Lytic and Total CMV proteins, Figure S7: Comparison of PBMC and BMMNC resident resting CD4+ T cell clustering analysis self-organising maps, Figure S8: Comparison of PBMC and BMMNC resident resting CD8+ T cell clustering analysis self-organising maps, Figure S9: Comparison of PBMC and BMMNC CMV-specific CD4+ T cell analysis. HCMV-specific T cells were identified and concatenated as described in the methods and Figures S3 and S4, Figure S10: Comparison of PBMC and BMMNC CMV-specific CD8+ T cell analysis, Figure S11: Inhibitory Receptor expression on IL-10-secreting CMV-specific T cells.

## Abbreviations

The following abbreviations are used in this manuscript:

APC	Antigen Presentation Cells
BM	Bone Marrow
BMMNC	Bone Marrow Mononuclear Cells
BTLA	B and T lymphocyte attenuator
CIR	Checkpoint Inhibitory Receptors
CMV	Cytomegalovirus
GPCR	G protein-coupled receptor
HCMV	Human Cytomegalovirus
HDACi	Histone deacetylase inhibitors
HVEM	Herpesvirus entry mediator
IBET	bromodomain and extra-terminal domain inhibitors
ICOS	Inducible Costimulator
ITIM	Immunoreceptor Tyrosine-based Inhibitory Motif
iTreg	Inducible T regulatory Cell
LAG-3	Lymphocyte Activation Gene 3
MCMV	Murine Cytomegalovirus
MMR	Measles Mumps and Rubella Vaccine
ORFs	Open Reading Frames
PB	Peripheral Blood
PBMCs	Peripheral Blood Mononuclear cells
SOM	Self Organising Maps
TCM	T central memory cells
TEM	T effector memory cells
TEMRA	T effector memory cells re-expressing CD45RA
Tfh	T follicular helper cells
TIGIT	T cell immunoglobulin and ITIM domain
Tregs	T regulatory Cells
tSNE	t-distributed stochastic neighbour embedding
WLSM	Weighted Least Squares Method
