# Supplementary material for: Distinct Immunological Landscapes of HCMV-Specific T Cells in Bone Marrow and Peripheral Blood
Source: Pathogens. 2025 Jul 22;14(8):722. doi: 10.3390/pathogens14080722 (PMC12389582; doi:10.3390/pathogens14080722)
Supplement: Supplementary file 1 [file pathogens-14-00722-s001.zip › pathogens-3746341-supplementary.pdf]

Jackson S.E. et al 2025 Supplementary Material

1 Supplementary Tables

Table S1: Major lymphocyte subsets antibody panel details

Antigen	Fluorochrome	Clone	Isotype	Cat. No.	Supplier
CD25	BUV395	2A3	IgG1κ	564034	BD Biosciences
CD56	BUV737	NCAM 16.2	IgG2bκ	612766	BD Biosciences
CD69	Pacific Blue	FN50	IgG1κ	310920	BioLegend
CD45	BV570	HI30	IgG1κ	304034	BioLegend
CD4	BV605	OKT4	IgG2bκ	317438	BioLegend
CD16	BV650	3G8	IgG1κ	302042	BioLegend
CD127	BV785	A019D5	IgG1κ	351330	BioLegend
CD105	FITC	43A3	IgG1κ	323204	BioLegend
CD14	PerCP-Cy5.5	M5E2	IgG2aκ	301824	BioLegend
CD8	PE	RPA-T8	IgG1κ	301008	BioLegend
CD34	PE-Dz594	581	IgG1κ	343534	BioLegend
HLA-DR	PE-Cy5	L243	IgG2aκ	307608	BioLegend
CD45RA	PE-Fire700	HI100	IgG2bκ	304172	BioLegend
CXCR4	PE-Cy7	12G5	IgG2aκ	306514	BioLegend
CD27	APC	O323	IgG1κ	302810	BioLegend
CD19	AxF700	SJ25C1	IgG1κ	363034	BioLegend
CD3	APC-Cy7	SK7	IgG1κ	344818	BioLegend
IgG1	BUV395	X40	Isotype	563547	BD Biosciences
IgG2b	BUV737	27-35	Isotype	612768	BD Biosciences
IgG1	Pacific Blue	MOPC-21	Isotype	400131	BioLegend
IgG1	BV570	MOPC-21	Isotype	400160	BioLegend
IgG2b	BV605	MPC-11	Isotype	400350	BioLegend
IgG1	BV650	MOPC-21	Isotype	400164	BioLegend
IgG1	BV785	MOPC-21	Isotype	400170	BioLegend
IgG1	FITC	MOPC-21	Isotype	400108	BioLegend
IgG2a	PerCP-Cy5.5	MOPC-173	Isotype	400251	BioLegend
IgG1	PE	MOPC-21	Isotype	400114	BioLegend
IgG1	PE-Dz594	MOPC-21	Isotype	400176	BioLegend
IgG2a	PE-Cy5	MOPC-173	Isotype	400218	BioLegend
IgG1	PE-Fire700	MOPC-21	Isotype	402414	BioLegend
IgG2a	PE-Cy7	MOPC-173	Isotype	400232	BioLegend
IgG1	APC	MOPC-21	Isotype	400122	BioLegend
IgG1	AxF700	MOPC-21	Isotype	400144	BioLegend
IgG1	APC-Cy7	MOPC-21	Isotype	400128	BioLegend

Abbrv: BV = Brilliant Violet; BUV = Brilliant UV; Dz = Dazzle; AxF = Alexa Fluor

Table S2: 37 Parameter Spectral Panel antibody clone and manufacturer details

Antigen	Fluorochrome	Clone	Isotype	Cat. No.	Supplier	Experiment Stage
CD107a	AxF647	H4A3	IgG1κ	300406	BioLegend	In overnight assay
CD14	FITC	M5E2	IgG2aκ	301804	BioLegend	Surface mix
CD19	SB Blue 615	LT19	IgG1κ	MCA1940SBB615	Bio-Rad	Surface mix
CD57	PE-Dz594	HNK-1	IgM κ	359620	BioLegend	Surface mix
CD45RA	PE-Fire700	HI100	IgG2bκ	304172	BioLegend	Surface mix
CD27	BUV496	O323	IgG1κ	751678	BD Biosciences	Surface mix
CD28	R718	CD28.2	IgG1κ	567492	BD Biosciences	Surface mix
CD69	Pacific Blue	FN50	IgG1κ	310920	BioLegend	Surface mix
CXCR4	BUV563	12G5	IgG2aκ	741400	BD Biosciences	Surface mix
CD25	BUV661	2A3	IgG1κ	741685	BD Biosciences	Surface mix
CD34	RB705	581	IgG1κ	570578	BD Biosciences	Surface mix
CD45	SB Yellow 720	F10-89-4	IgG2aκ	MCA87SBY720	Bio-Rad	Surface mix
CD127	APC-Fire810	A019D5	IgG2aκ	351374	BioLegend	Surface mix
TIGIT	APC	REA1004	rhIgG1	130-116-815	Miltenyi Biotec	Surface mix
ICOS	BUV615	DX29	IgG1κ	751092	BD Biosciences	Surface mix
Tim-3	BUV805	F38-2E2	IgG1κ	368-3109-42	Thermo Fisher	Surface mix
BTLA	BV480	J168-540	IgG1κ	746759	BD Biosciences	Surface mix
2B4	APC-Vio770	REA112	rhIgG1	130-123-460	Miltenyi Biotec	Surface mix
LAG-3	BV750	11C3C65	IgG1κ	369351	BioLegend	Surface mix
CD2	RB545	RPA-2.10	IgG1κ	756590	BD Biosciences	Surface mix
GITR	PE-Cy7	108-17	IgG2aκ	371224	BioLegend	Surface mix
CTLA-4	APC-Cy7	L3D10	IgG1κ	349942	BioLegend	Surface mix
KLRG1	PerCP-Fire806	SA231A2	IgG2aκ	367748	BioLegend	Surface mix
PD-1	BV421	EH12.2H7	IgG1κ	329920	BioLegend	Intracellular mix
TNFα	BUV395	Mab11	IgG1κ	563996	BD Biosciences	Intracellular mix
IL-2	BUV737	MQ1-17H12	Rat IgG2aκ	612836	BD Biosciences	Intracellular mix
IL-10	BV711	JES3-9D7	Rat IgG1κ	564050	BD Biosciences	Intracellular mix
CD39	RB744	Tu66	IgG2bκ	570637	BD Biosciences	Intracellular mix
CD8	BV570	RPA-T8	IgG1κ	301038	BioLegend	Intracellular mix
CD4	BV605	OKT4	IgG2bκ	317438	BioLegend	Intracellular mix
CD3	BV650	OKT3	IgG2aκ	317324	BioLegend	Intracellular mix
IFNγ	BV786	4S.B3	IgG1κ	563731	BD Biosciences	Intracellular mix
CD40L	PerCP-Fire780	24-31	IgG1κ	310859	BioLegend	Intracellular mix
OX-40	PE	Ber-ACT35	IgG1κ	350004	BioLegend	Intracellular mix
4-1BB	PE-Cy5	4B4-1	IgG1κ	309808	BioLegend	Intracellular mix
HLA-DR	PE-Fire 810	L243	IgG2aκ	307683	BioLegend	Intracellular mix

Abbrv: BV = Brilliant Violet; BUV = Brilliant UV; Dz = Dazzle; AxF = Alexa Fluor; SB = Star Bright.

Table S3: High Dimensional Flowcytometry analysis FlowSOM algorithm runs FlowJo v10.10

Concatenated FCS file	Subset	Parameters group	Map ID	Map on
CD4 CMV+ PB BM US concat_1	CD4	Functional (5 parameters)	PBVU	-
	IE1		EBLL	PBVU
	US28		TAZW	PBVU
	Unstim		WGXI	PBVU
	IE1 PBMC		QJPC	PBVU
	IE1 BMMC		KU6L	PBVU
	US28 PBMC		3RYC	PBVU
	US28 BMMC		KPD7	PBVU
	Unstim PBMC		CH1K	PBVU
	Unstim BMMC		NREH	PBVU
CD4 CMV+ PB BM US concat_1	CD4	Activation (9 parameters)	DBJ2	-
	IE1		HTCO	DBJ2
	US28		GBRS	DBJ2
	Unstim		0PSO	DBJ2
	IE1 PBMC		IXXA	DBJ2
	IE1 BMMC		ZKCA	DBJ2
	US28 PBMC		RJIH	DBJ2
	US28 BMMC		1U1B	DBJ2
	Unstim PBMC		L9O6	DBJ2
	Unstim BMMC		J1KC	DBJ2
CD4 CMV+ PB BM US concat_1	CD4	Checkpoint (11 parameters)	BCEO	-
	IE1		KBCF	BCEO
	US28		FFOW	BCEO
	Unstim		VL25	BCEO
	IE1 PBMC		8IIG	BCEO
	IE1 BMMC		EJK4	BCEO
	US28 PBMC		RA70	BCEO
	US28 BMMC		4Q9U	BCEO
	Unstim PBMC		6MTJ	BCEO
	Unstim BMMC		Y7P3	BCEO
CD8 CMV+ BM PM US concat file_1	CD8	Functional (5 parameters)	C7XQ	-
	IE1		ZJAC	C7XQ
	US28		0ZSQ	C7XQ
	Unstim		HNBL	C7XQ
	IE1 PBMC		IO4T	C7XQ

	IE1 BMMC		MYAG	C7XQ
	US28 PBMC		PYNV	C7XQ
	US28 BMMC		NNB4	C7XQ
	Unstim PBMC		TR40	C7XQ
	Unstim BMMC		SYPN	C7XQ
CD8 CMV+ BM PM US concat file_1	CD8	Activation (9 parameters)	NFQ3	-
	IE1		VDTD	NFQ3
	US28		2L9K	NFQ3
	Unstim		LS4V	NFQ3
	IE1 PBMC		9BNC	NFQ3
	IE1 BMMC		B7AM	NFQ3
	US28 PBMC		MK1Y	NFQ3
	US28 BMMC		B80F	NFQ3
	Unstim PBMC		CRS8	NFQ3
	Unstim BMMC		QFNT	NFQ3
CD8 CMV+ BM PM US concat file_1	CD8	Checkpoint (11 parameters)	99O4	-
	IE1		73G8	99O4
	US28		70X4	99O4
	Unstim		Y5YT	99O4
	IE1 PBMC		VQBJ	99O4
	IE1 BMMC		D4QJ	99O4
	US28 PBMC		MNTT	99O4
	US28 BMMC		75Z7	99O4
	Unstim PBMC		6T6Y	99O4
	Unstim BMMC		LKKK	99O4
POS ctrl positives_1.fcs	CD4	Functional (5 parameters)	L0KI	-
	POS		Q83A	L0KI
	Unstim		QXC3	L0KI
	POS PBMC		KCOD	L0KI
	POS BMMC		O7QR	L0KI
	Unstim PBMC		9HWO	L0KI
	Unstim BMMC		7W6M	L0KI
POS ctrl positives_1.fcs	CD4	Activation (9 parameters)	RBV8	-
	POS		BPAB	RBV8
	Unstim		6GN8	RBV8
	POS PBMC		IMM3	RBV8
	POS BMMC		1O93	RBV8
	Unstim PBMC		6I7E	RBV8

	Unstim BMMC		ETUB	RBV8
POS ctrl positives_1.fcs	CD4	Checkpoint (11 parameters)	B4OM	-
	POS		SLXF	B4OM
	Unstim		ILYB	B4OM
	POS PBMC		JU21	B4OM
	POS BMMC		KUBE	B4OM
	Unstim PBMC		NWTP	B4OM
	Unstim BMMC		VL0U	B4OM

Supplementary Figures

Figure S1

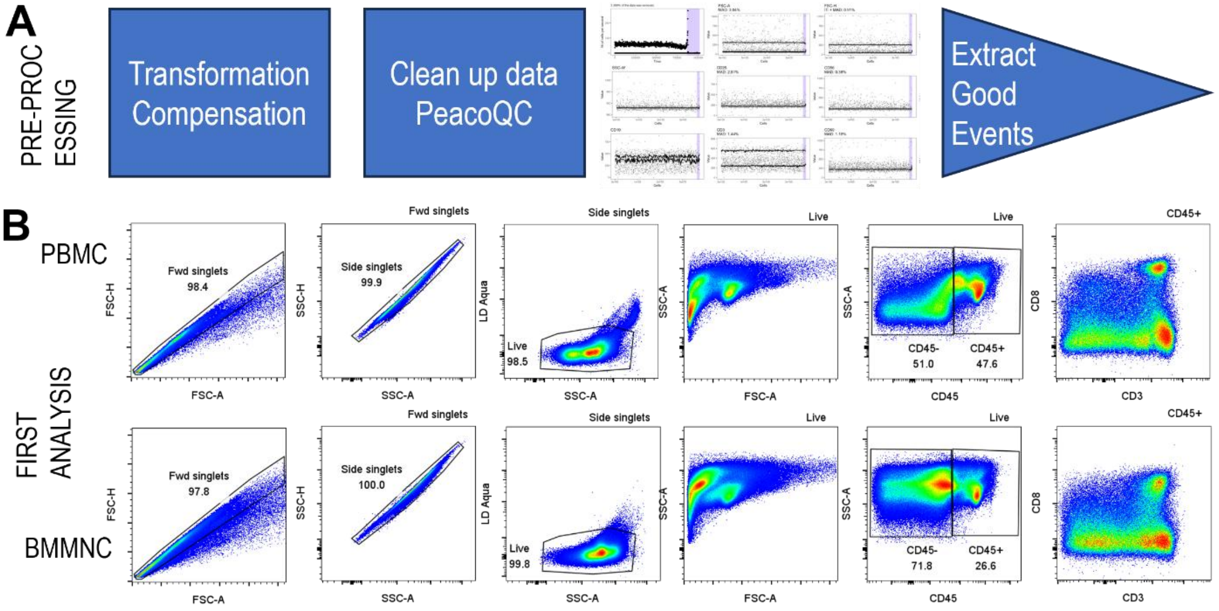

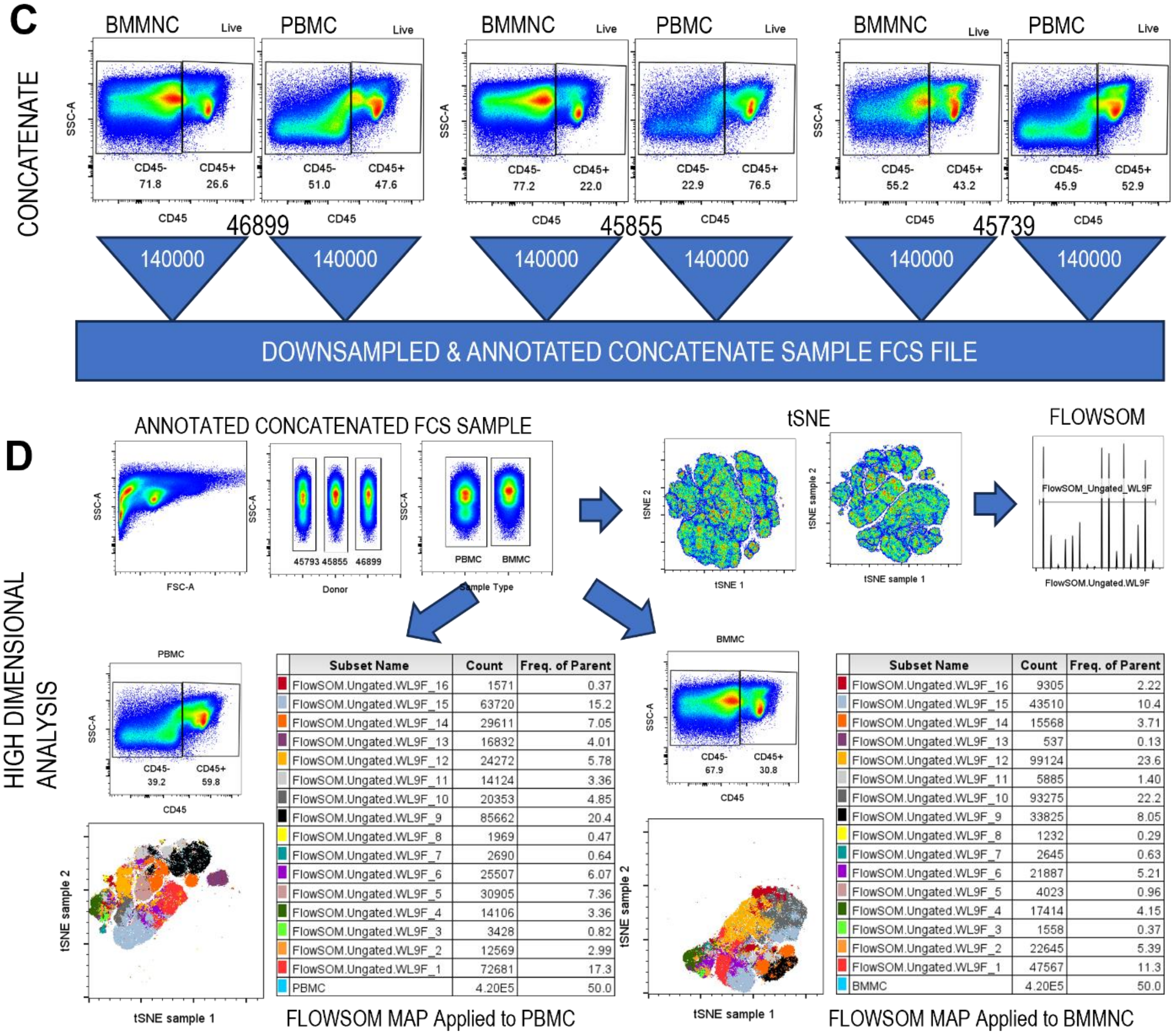

Subset Name	Count	Freq. of Parent
FlowSOM.Ungated.WL9F_16	1571	0.37
FlowSOM.Ungated.WL9F_15	63720	15.2
FlowSOM.Ungated.WL9F_14	29611	7.05
FlowSOM.Ungated.WL9F_13	16832	4.01
FlowSOM.Ungated.WL9F_12	24272	5.78
FlowSOM.Ungated.WL9F_11	14124	3.36
FlowSOM.Ungated.WL9F_10	20353	4.85
FlowSOM.Ungated.WL9F_9	85662	20.4
FlowSOM.Ungated.WL9F_8	1969	0.47
FlowSOM.Ungated.WL9F_7	2690	0.64
FlowSOM.Ungated.WL9F_6	25507	6.07
FlowSOM.Ungated.WL9F_5	30905	7.36
FlowSOM.Ungated.WL9F_4	14106	3.36
FlowSOM.Ungated.WL9F_3	3428	0.82
FlowSOM.Ungated.WL9F_2	12569	2.99
FlowSOM.Ungated.WL9F_1	72681	17.3
PBMC	4.20E5	50.0

Subset Name	Count	Freq. of Parent
FlowSOM.Ungated.WL9F_16	9305	2.22
FlowSOM.Ungated.WL9F_15	43510	10.4
FlowSOM.Ungated.WL9F_14	15568	3.71
FlowSOM.Ungated.WL9F_13	537	0.13
FlowSOM.Ungated.WL9F_12	99124	23.6
FlowSOM.Ungated.WL9F_11	5885	1.40
FlowSOM.Ungated.WL9F_10	93275	22.2
FlowSOM.Ungated.WL9F_9	33825	8.05
FlowSOM.Ungated.WL9F_8	1232	0.29
FlowSOM.Ungated.WL9F_7	2645	0.63
FlowSOM.Ungated.WL9F_6	21887	5.21
FlowSOM.Ungated.WL9F_5	4023	0.96
FlowSOM.Ungated.WL9F_4	17414	4.15
FlowSOM.Ungated.WL9F_3	1558	0.37
FlowSOM.Ungated.WL9F_2	22645	5.39
FlowSOM.Ungated.WL9F_1	47567	11.3
BMMC	4.20E5	50.0

Figure S1. Major lymphocyte subsets workflow and representative flow cytometer plots. The pre-processing analysis workflow of the LSR Fortessa acquired FCS files is illustrated (A) – a compensation matrix was generated from single colour controls run with the samples in FlowJo v10.10 using the autospill algorithm and then the individual parameters were transformed to ensure that the staining data is optimized prior to a quality control algorithm PeacoQC being run on all FCS files. The extracted events were then analysed with conventional gating strategy to identify the live cells, representative data from donor 46899 is shown (B). The live cells from the first analysis for all 3 donors and sample types were annotated, down sampled and concatenated (C). The resulting FCS file is illustrated (D) with the resulting dimensionality reduction (tSNE) and clustering algorithm (FlowSOM plugin map ID WLSF) results illustrated – finally the distribution of the 16 clusters from the FlowSOM map applied to the PBMC and BMMNC cells are shown overlayed on the tSNE.

Figure S2

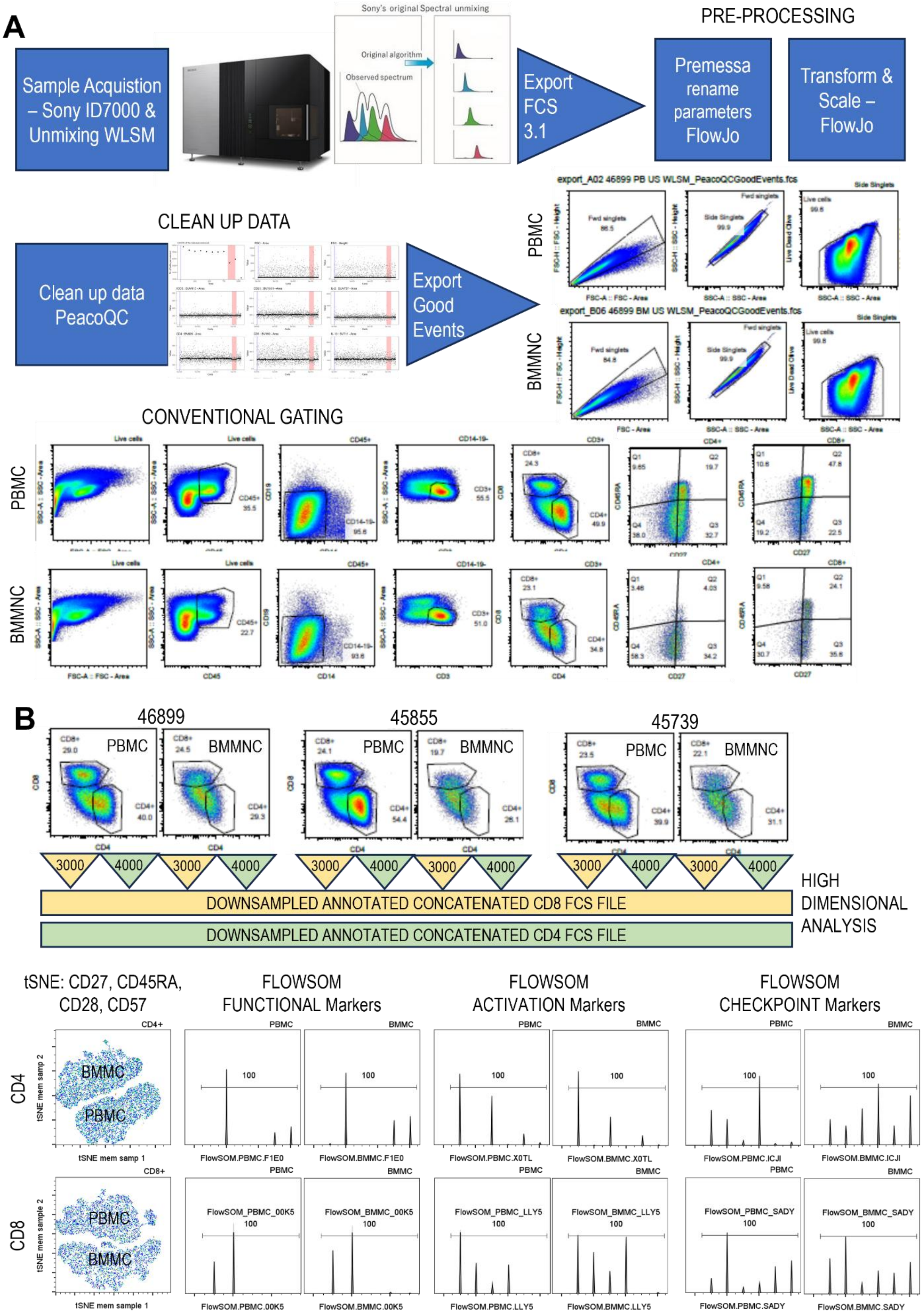
A
Sample Acquistion – Sony ID7000 & Unmixing WLSM
Sony's original Spectral unmixing
Original algorithm
Observed spectrum
Export FCS 3.1
PRE-PROCESSING
Premessa rename parameters FlowJo
Transform & Scale – FlowJo
CLEAN UP DATA
Clean up data PeacoQC
Export Good Events
export_A02 46899 PB US WLSM_PeacoQCGoodEvents.fcs
export_B06 46899 BM US WLSM_PeacoQCGoodEvents.fcs
PBMC
BMMNC
Fwd singlets
Side Singlets
Live cells
CONVENTIONAL GATING
B
46899
45855
45739
PBMC
BMMNC
3000
4000
DOWNSAMPLED ANNOTATED CONCATENATED CD8 FCS FILE
DOWNSAMPLED ANNOTATED CONCATENATED CD4 FCS FILE
HIGH DIMENSIONAL ANALYSIS
tSNE: CD27, CD45RA, CD28, CD57
FLOWSOM FUNCTIONAL Markers
FLOWSOM ACTIVATION Markers
FLOWSOM CHECKPOINT Markers
CD4
CD8
BMMC
PBMC
FlowSOM.PBMC.F1E0
FlowSOM.BMMC.F1E0
FlowSOM.PBMC.X0TL
FlowSOM.BMMC.X0TL
FlowSOM.PBMC.ICJI
FlowSOM.BMMC.ICJI
FlowSOM.PBMC.00K5
FlowSOM.BMMC.00K5
FlowSOM.PBMC.LLY5
FlowSOM.BMMC.LLY5
FlowSOM.PBMC.SADY
FlowSOM.BMMC.SADY

Supplementary Figure S2. Resting cells analysis spectral workflow and representative gating. The workflow for sample acquisition using a 5-laser configured Sony ID7000 Spectral Cell Analyser is illustrated (A) – samples were acquired in standardized mode with spectral unmixing and autofluorescence subtraction performed by the Sony system software using a Weighted Least Squares Method (WLSM) algorithm. The unmixed FCS 3.1 files were exported for further downstream analysis using FlowJo v10.10, the individual FCS files were pre-processed using the Premessa plugin to remove square brackets ([ ]) from the parameter names and then the individual parameters were transformed to ensure that the staining data is optimized. A quality control algorithm (PeacoQC) was run to ensure only good events were exported and analysed. Representative data from donor 46899 illustrates the conventional gating strategy used to identify CD4+ and CD8+ T cells for further analysis, forward and side singlets were identified, followed by live cells and then CD45 positive, CD14 and CD19 negative cells were gated for CD3+ and then CD4 vs CD8 gate – also shown are the CD27 vs CD45RA memory population dot plots for CD4+ and CD8+ T cells from both PBMC and BMMNC samples. CD4+ or CD8+ T cells from PBMC and BMMNC and all three donors were annotated, down sampled and concatenated for multi-dimensional analysis as illustrated (B). On both the CD8 and CD4 FCS files a tSNE using the 4 memory and differentiation phenotype markers (CD27, CD45RA, CD28, CD57) were run with the results shown with the BMMNC and PBMC samples identified. Three different FlowSOM clustering maps were run for both the total CD4 and CD8 Files and the resulting clusters from these maps being applied to the PBMC and BMMNC samples are shown as histograms for each compartment. The different FlowSOM Map IDs were for CD4+ Functional (5 markers map ID – F1E0), Activation (9 markers map ID – X0TL) and Checkpoint (11 markers map ID – ICJI). The FlowSOM Map IDs for the CD8+ analysis were Functional (Map ID – 00K5), Activation (Map ID – LLY5) and Checkpoint (Map ID – SADY).

Figure S3

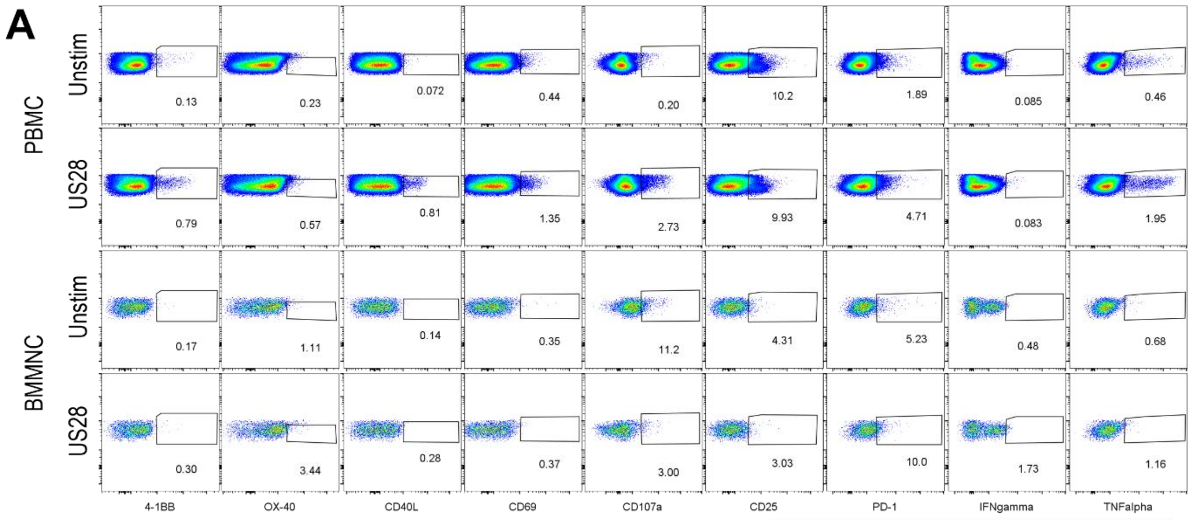

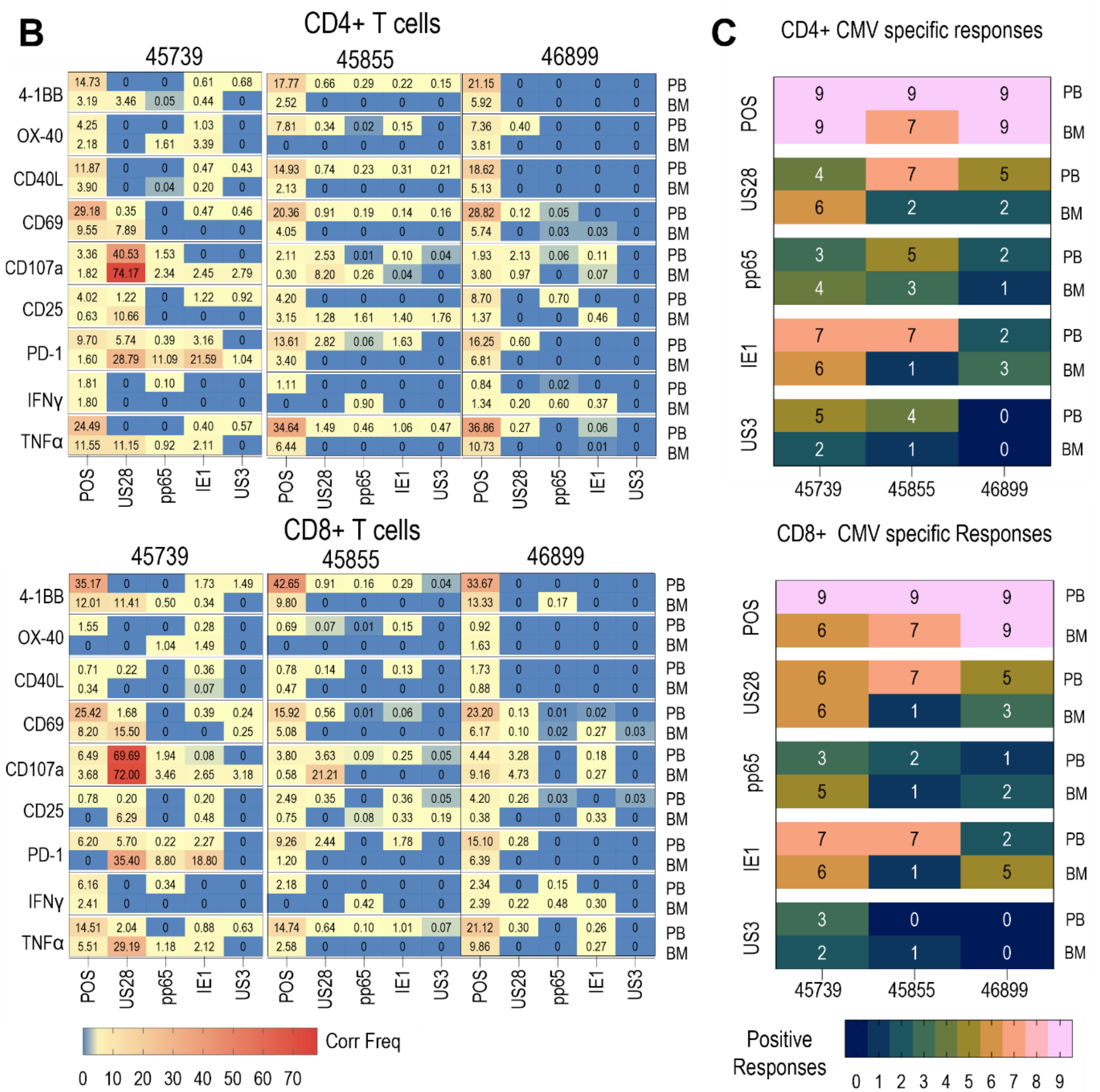

Supplementary Figure S3. Determining HCMV specific T cell responses following spectral flow cytometry. Representative dots plots from the CD4+ T cells for donor 45855 for unstimulated and US28 stimulation in the PBMC and BMMNC for nine different activation associated markers – 4-1BB, OX-40, CD40L, CD69, CD107a, CD25, PD-1, IFNγ and TNFα are shown (A). The responses for all three donors CD4+ and CD8+ T cells are summarized in heatmaps (B) for all stimulations following background subtraction of the unstimulated PBMC or BMMNC control. The number of activation markers out of 9 that were above the 0.05 threshold are summarized as a heatmap (C). A positive response to HCMV peptide stimulation by either the PBMC or BMMNC for each donor was determined as a minimum of five out of nine responding markers above threshold. Using this method the responses to US28 and IE1 stimulation were selected for further analysis of the HCMV specific response as all three donors had a positive response to these proteins in either the PBMC or BMMNC compartments.

Figure S4

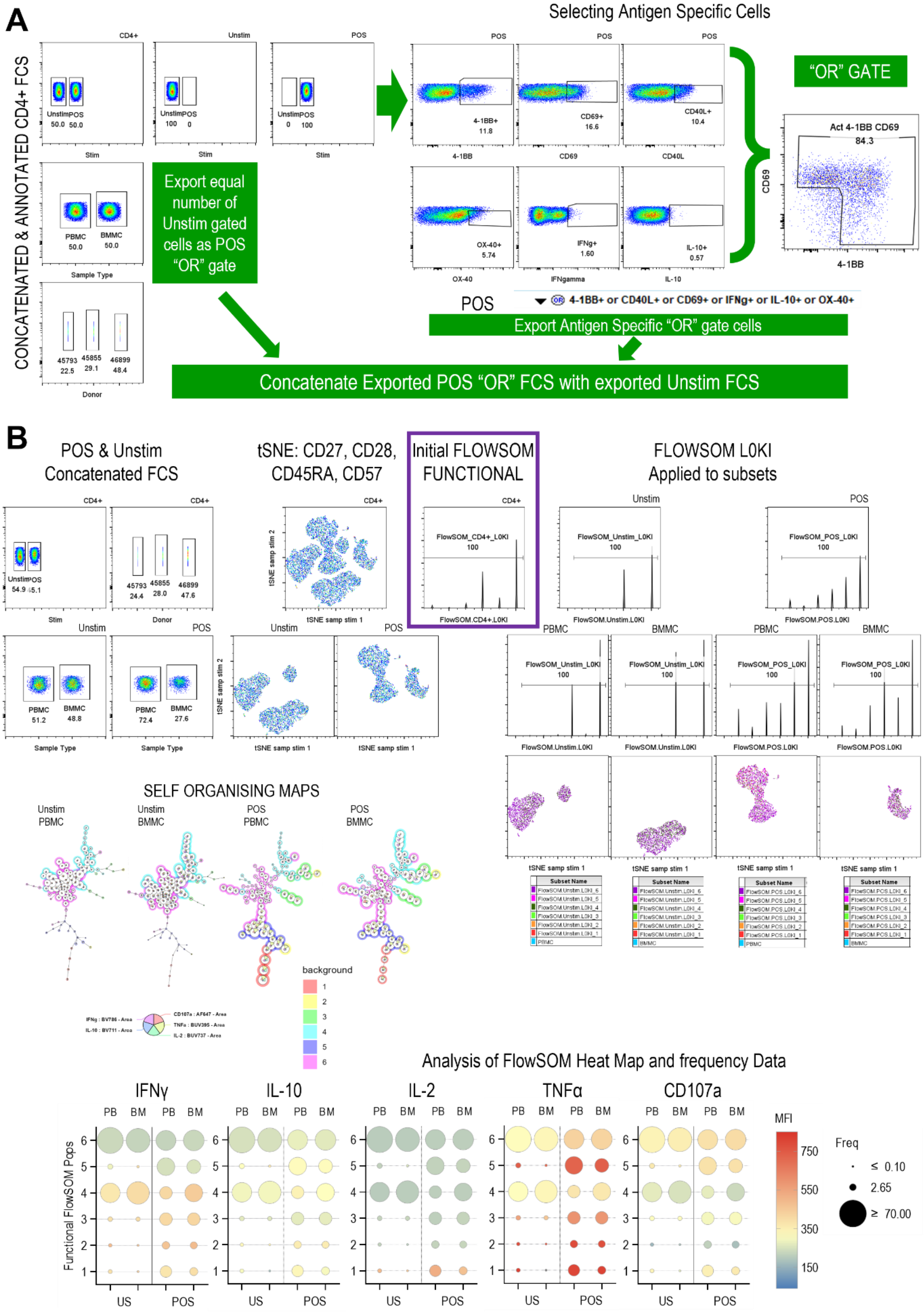
A
Selecting Antigen Specific Cells
CONCATENATED & ANNOTATED CD4+ FCS
"OR" GATE
Export equal number of Unstim gated cells as POS "OR" gate
Act 4-1BB CD69
84.3
POS
4-1BB+ or CD40L+ or CD69+ or IFNg+ or IL-10+ or OX-40+
Export Antigen Specific "OR" gate cells
Concatenate Exported POS "OR" FCS with exported Unstim FCS
B
POS & Unstim Concatenated FCS
tSNE: CD27, CD28, CD45RA, CD57
Initial FLOWSOM FUNCTIONAL
FLOWSOM L0KI Applied to subsets
SELF ORGANISING MAPS
Unstim PBMC
Unstim BMMC
POS PBMC
POS BMMC
background
Analysis of FlowSOM Heat Map and frequency Data
IFNγ
IL-10
IL-2
TNFα
CD107a
Functional FlowSOM Pops
US
POS
MFI
Freq
≤ 0.10
2.65
≥ 70.00

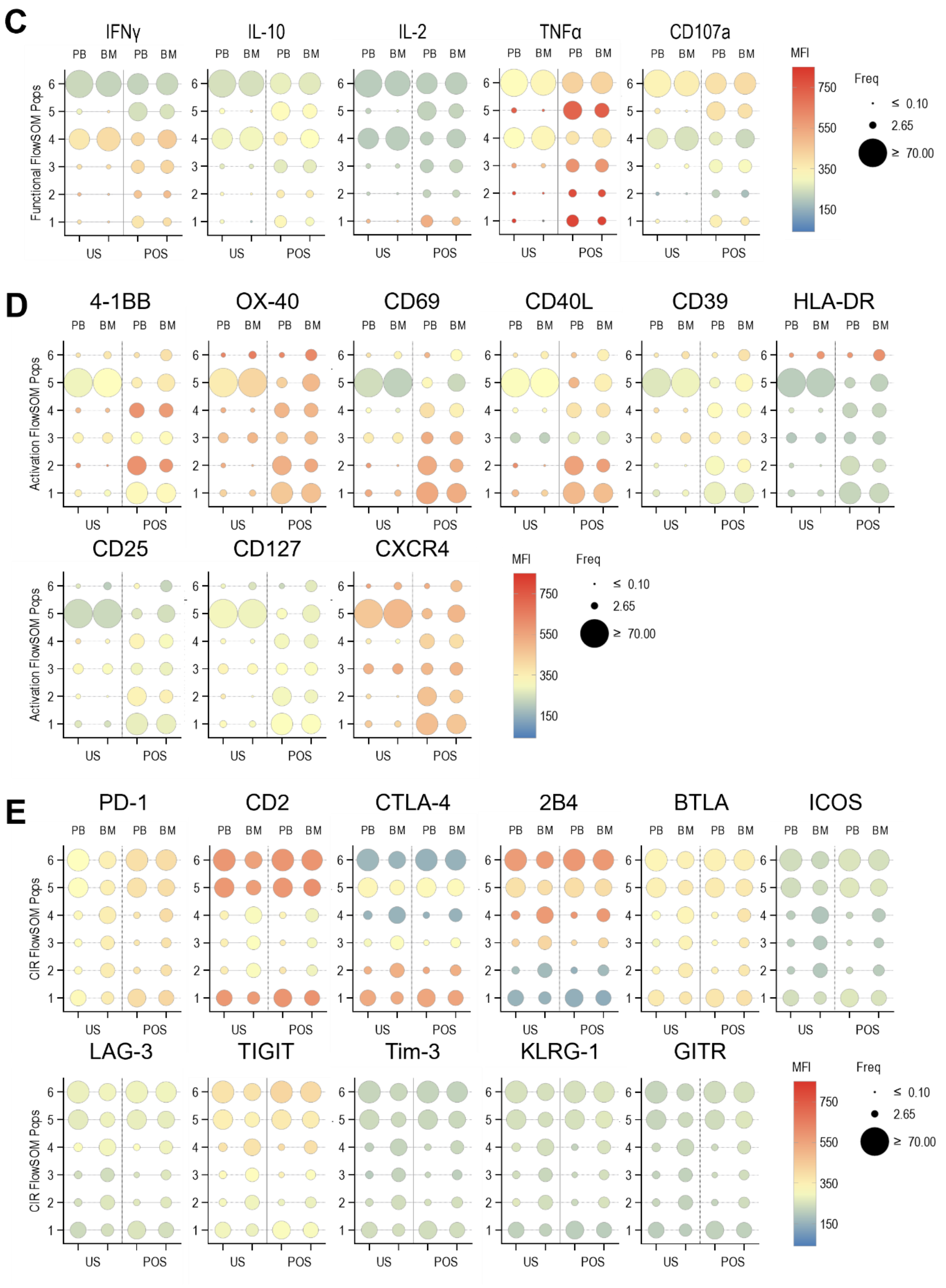

Supplementary Figure S4. Workflow for selecting antigen specific T cells and subsequent analysis using the CD4+ Positive control as representative example. In order to analyse the antigen specific T cell response to resting cells from each tissue compartment a Boolean Logic "OR" gate was used to identify activated cells. Shown

are the results from the CD4+ Unstimulated and Positive Control concatenated FCS file (A), the parameters included in the "OR" gate (4-1BB, CD69, CD40L, OX-40, IFNγ+ and IL-10+) are illustrated and a dot plot of 4-1BB v CD69 expression of the compiled OR gate is also shown. The subsequent export and then re-concatenation process, described in methods section *2.7.3*, to generate the final analysis file is then illustrated. The multi-dimensional analysis sequence using the generated CD4+ POS "OR" gate concatenated file is illustrated (B), with the tSNE (CD27, CD28, CD45RA, CD57, sample time and stimulation parameters) shown, next the typical outputs of data from the clustering analysis are shown for the Functional FlowSOM (CD107a, TNFα, IL-2, IL-10 and IFNγ – Map ID L0KI) initially run on the total file and then the map was applied to the different stimulation and sample type subsets – overlays of the 6 clustering populations for the different samples data and the self-organizing maps (SOM) are shown. The data generated from the FlowSOM analysis – distribution of the frequency of events per FlowSOM population alongside the intensity of staining (MFI) as a heatmap colour for each bubble are shown as individual plots for the five parameters. The results from the full analysis of the CD4+ POS control FCS file for the three different FlowSOM runs are shown as individual parameter bubble plots showing the frequency of distribution of the 6 population clusters for the PBMC (PB) and BMMNC (BM) samples from all three donors for the unstimulated and positive control stimulation. The bubble plots are coloured according to the intensity of expression of each marker on the scale illustrated in the heatmap bar in the key to the graphs. The results from the Functional FlowSOM (C), Activation marker FlowSOM (D) and Checkpoint Inhibitor Receptors (CIR) FlowSOM (E) are illustrated.

Figure S5

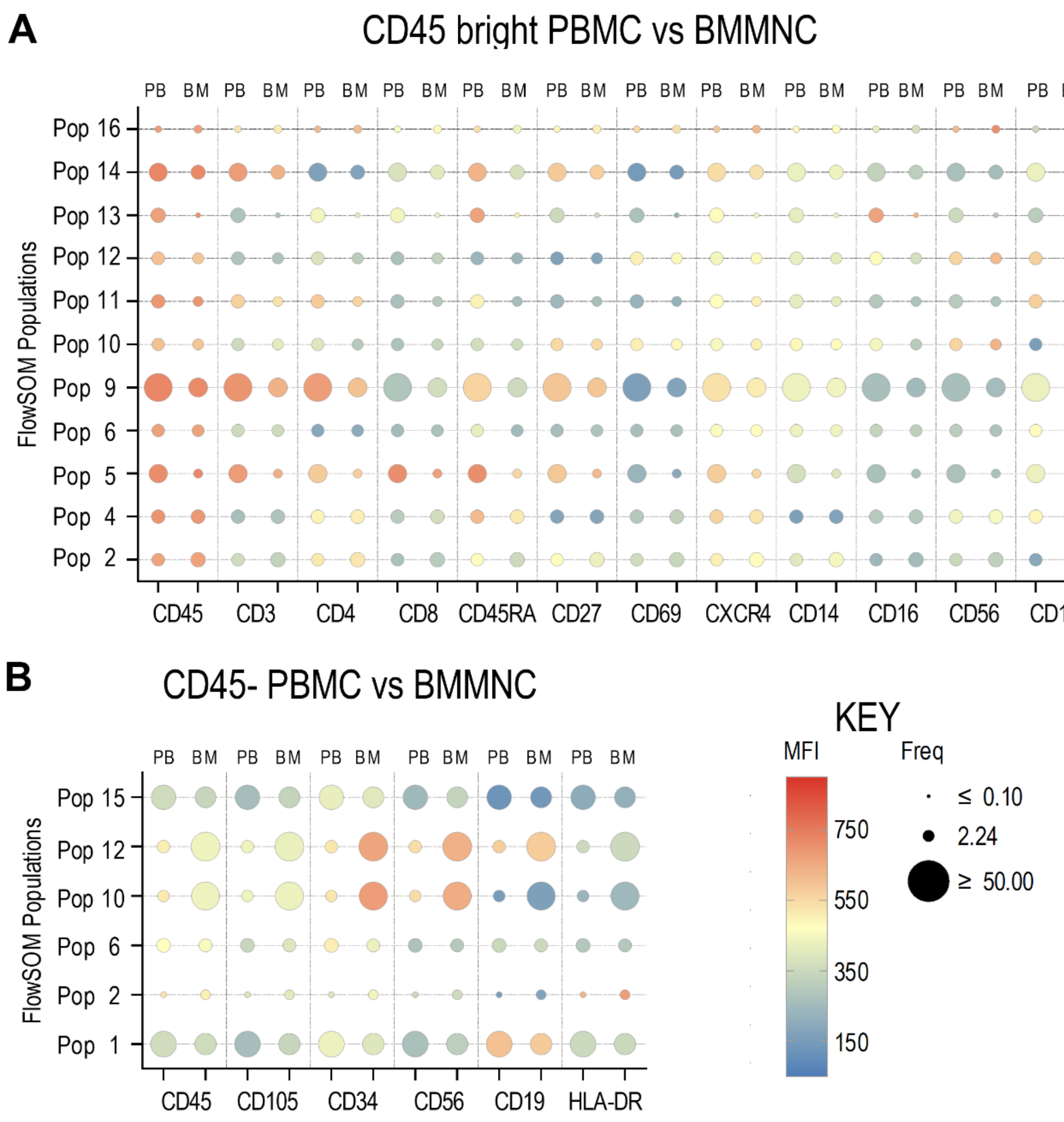

Supplementary Figure S5. Frequency and marker expression intensity of the CD45 positive and CD45 negative analysis of the major cellular populations clustering analysis. The PBMC and BMMNC samples from the three donors were stained with a panel of antibodies to identify major lymphocyte and non-lymphocyte cells present in the paired samples by flow cytometry. The resulting data files were analysed and then concatenated together (workflow shown in figure S1). A FlowSOM clustering algorithm was performed on the concatenated file and selected results are summarized for the maps of the CD45+ PBMC (PB) and BMMNC (BM) (A) and CD45- cells (B) are shown. The size of the bubbles in the graphs indicates the frequency of the distribution of each sample between the FlowSOM clusters and the heatmap colour shows the intensity of each marker's expression as generated by the FlowSOM algorithm (shown in Key). Related FlowSOM maps are shown in Figure 1.

Figure S6

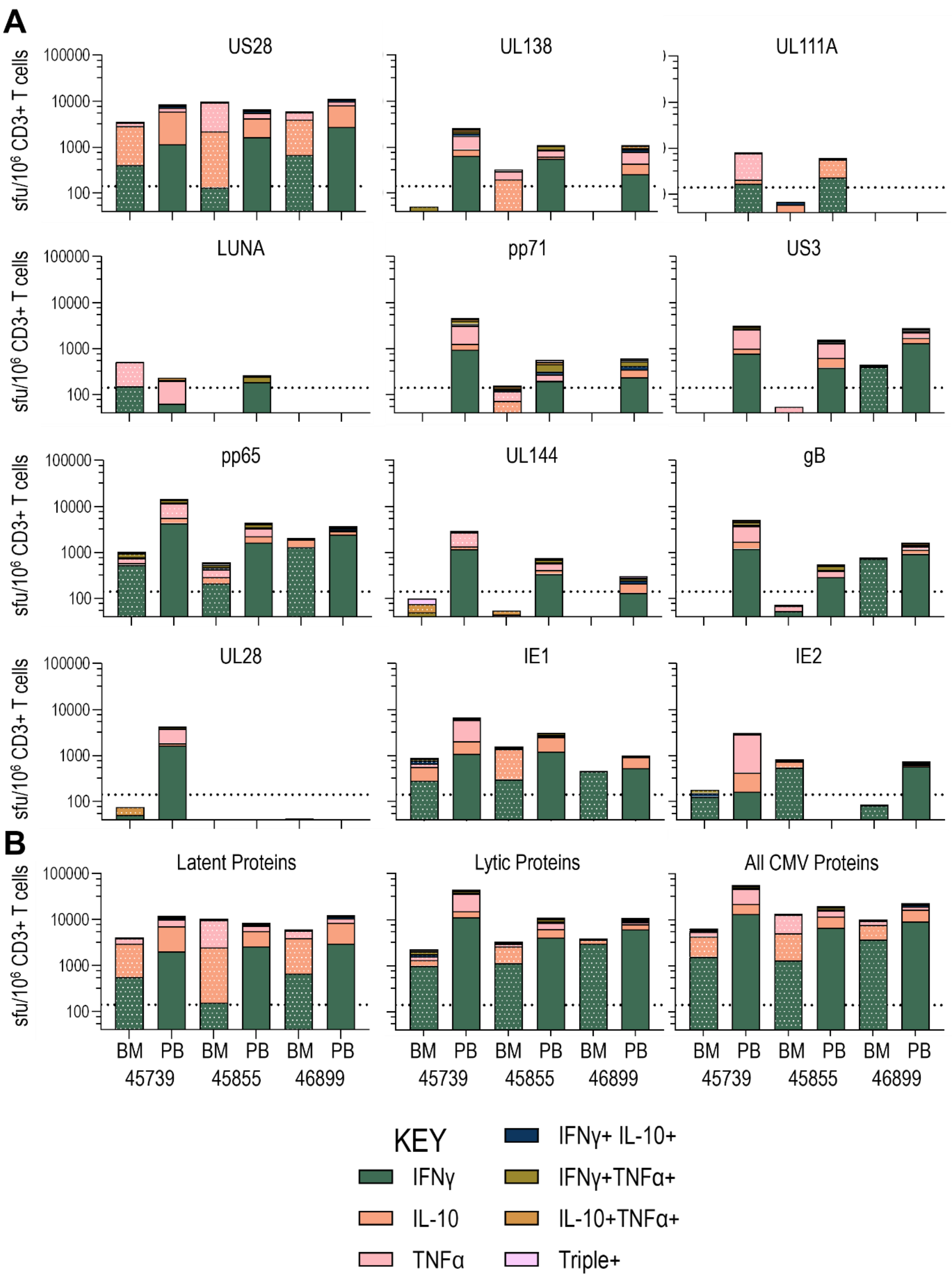
A
US28
UL138
UL111A
LUNA
pp71
US3
pp65
UL144
gB
UL28
IE1
IE2
B
Latent Proteins
Lytic Proteins
All CMV Proteins
sfu/10⁶ CD3+ T cells
100000
10000
1000
100
BM
PB
45739
45855
46899
KEY
IFNγ
IL-10
TNFα
IFNγ+ IL-10+
IFNγ+TNFα+
IL-10+TNFα+
Triple+

Supplementary Figure S6. Magnitude and cytokine secreting composition of PBMC and BMMNC from the three donors for the individual HCMV, Latent, Lytic and Total CMV proteins.

The cytokine secreting response of CD3+ T cells from BMMNC (BM) and PBMC (PB) following stimulation by 12 HCMV protein overlapping peptide pools were measured in the three paired donors using a triple fluorospot method. The results have been converted into spot forming units per million CD3+ T cells (sfu/10^6 CD3+ T cells) with background counts for each cytokine subtracted. The magnitude of the total cytokine response (sum of single, dual and triple secreting cells) to stimulation by each individual HCMV protein (A) are shown by the size of the bar for each donor and sample type – the dotted line represents the positive threshold for the combined cytokine response following stimulation. The colours in the stacked bars represents the IFNγ, IL-10, TNFα and dual and triple secreting cells present in response to stimulation. The cumulative response to 4 Latent (US28, UL138, UL111A and LUNA) and 8 Lytic (pp71, US3, pp65, UL144, gB, UL28, IE1 and IE2) proteins as well as the response to all 12 proteins are also shown as stacked bar graphs for each donor (B).

Figure S7

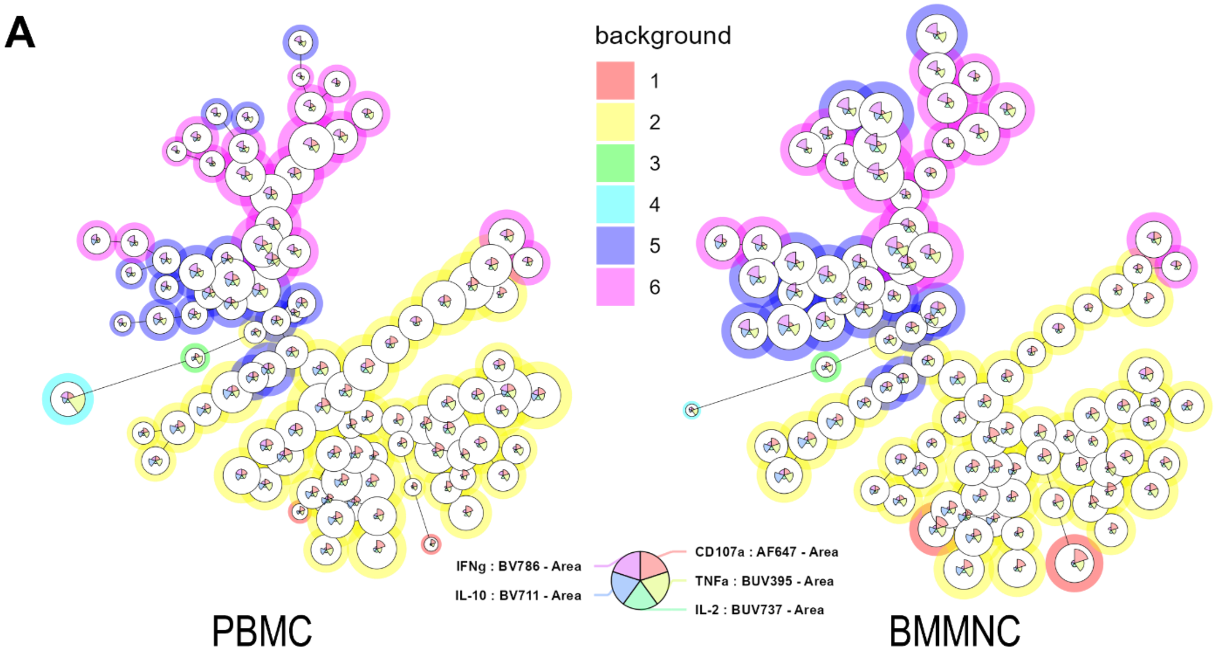

B

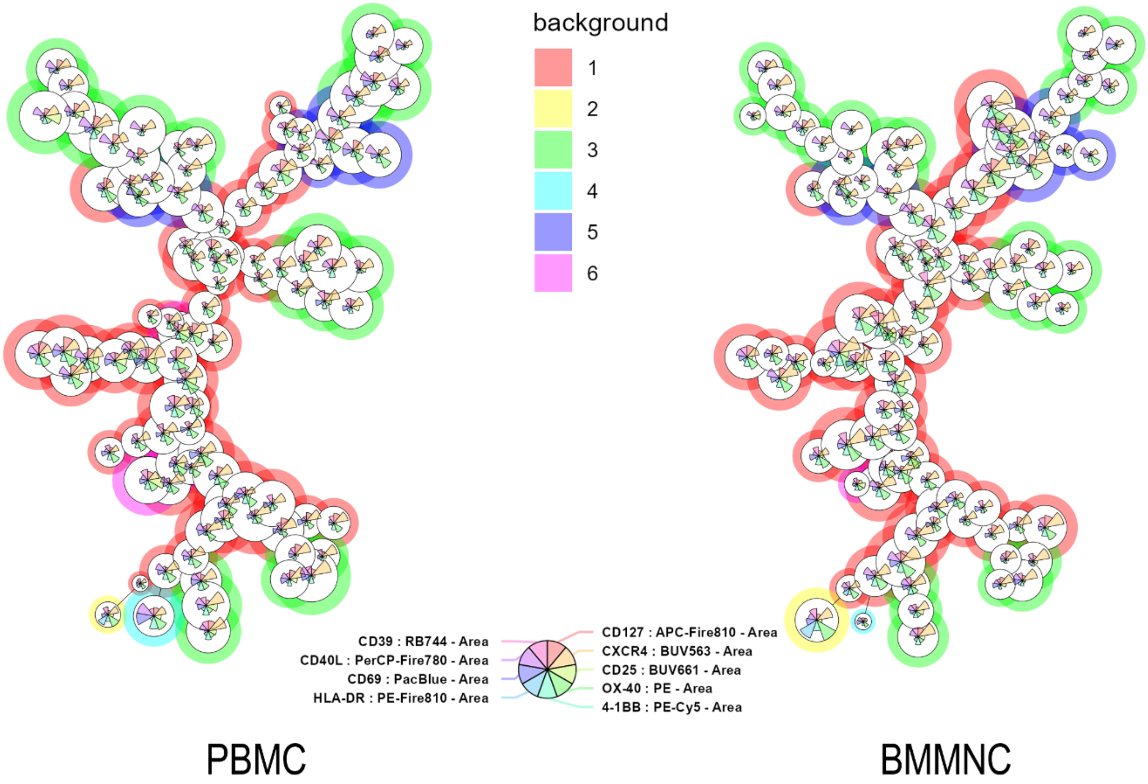

C

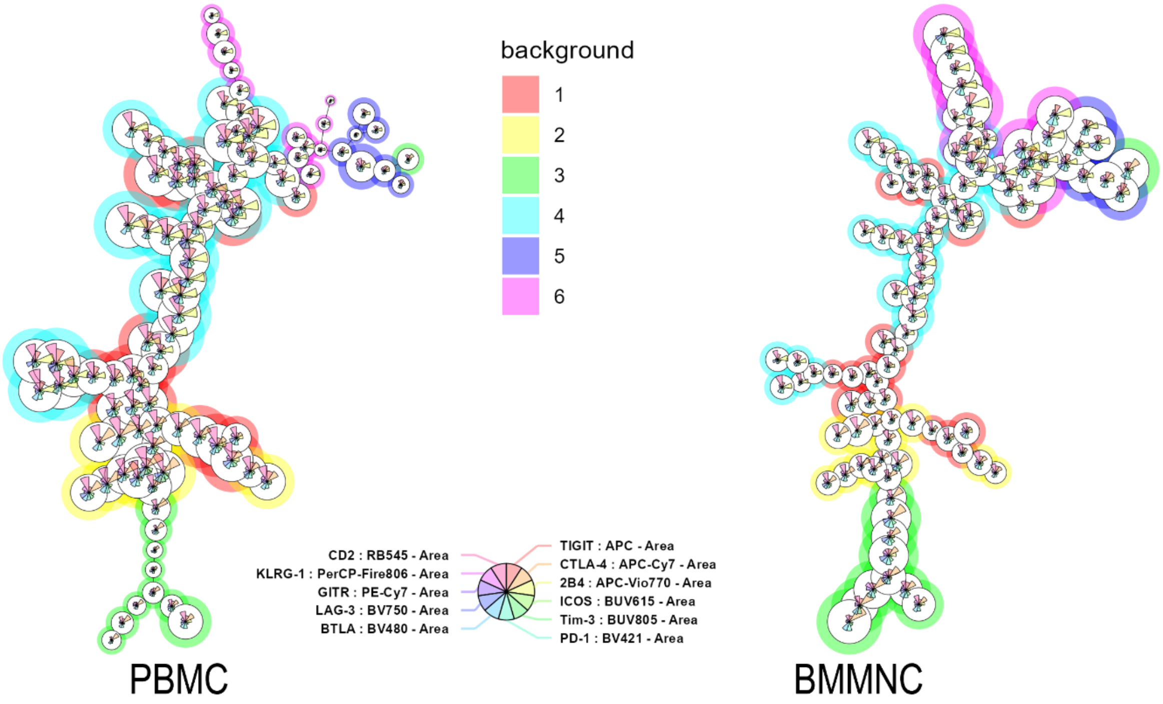

Supplementary Figure S7. Comparison of PBMC and BMMNC resident resting CD4+ T cell clustering analysis self-organising maps. Gated CD4+ T cells from the three donors were concatenated and analysed as outlined in the methods and Figure S2. Three separate clustering FlowSOM algorithms were performed and shown here are

the self-organising maps from the PBMC and BMMNC applied clusters for the Functional FlowSOM (A) the Activation markers FlowSOM (B) and CIR FlowSOM (C).

Figure S8

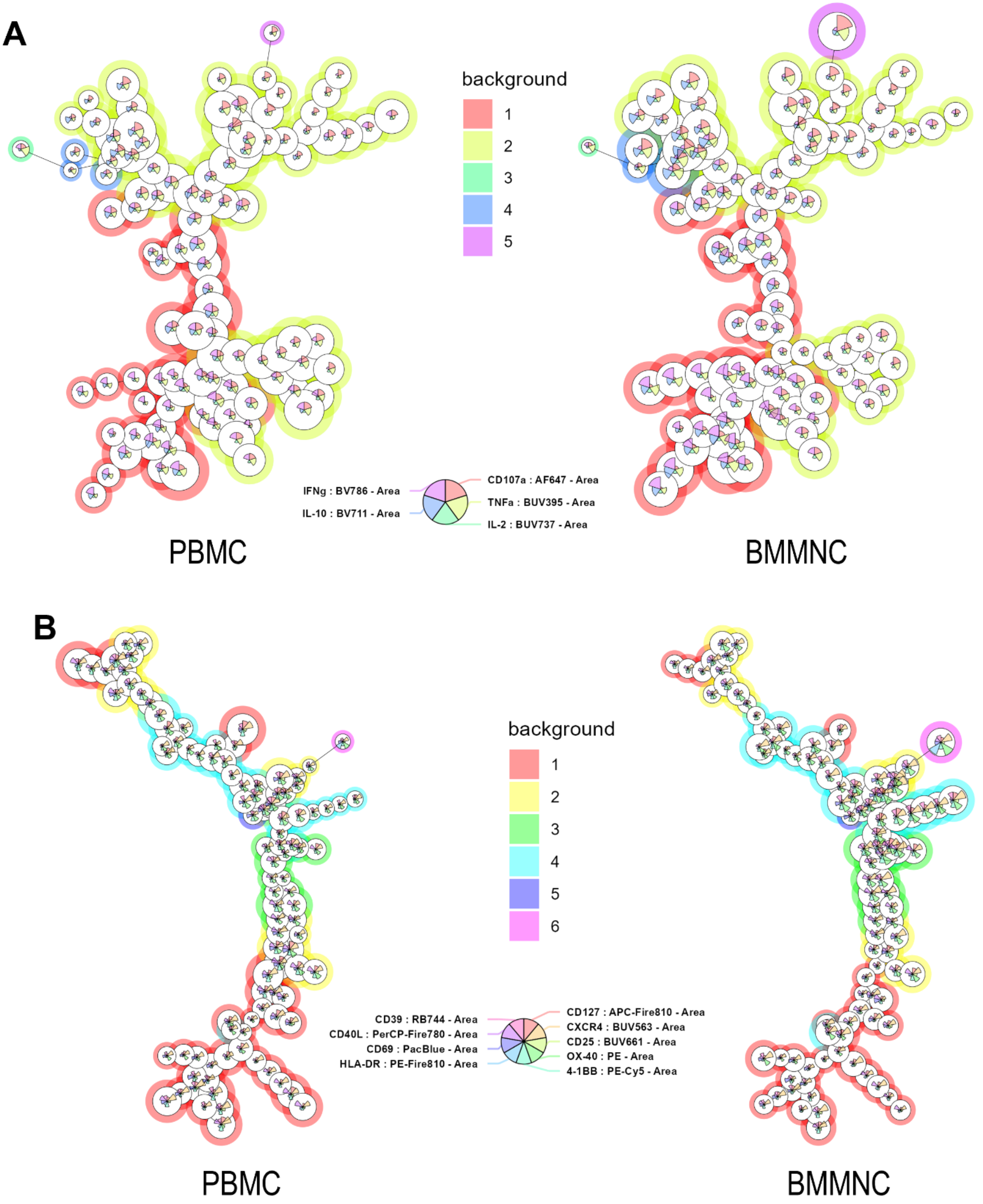

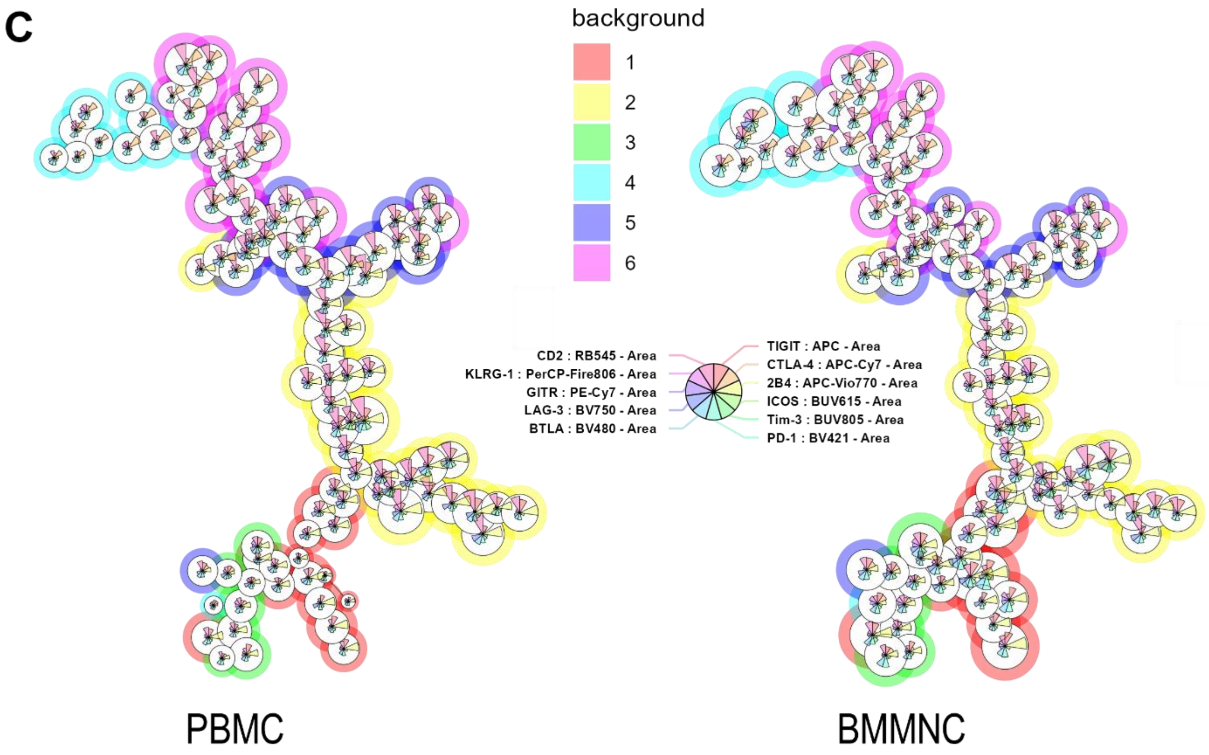

Supplementary Figure S8. Comparison of PBMC and BMMNC resident resting CD8+ T cell clustering analysis self-organising maps. Gated CD8+ T cells from the three donors were concatenated and analysed as previously described. Three separate clustering FlowSOM algorithms were performed and shown here are the self-organising maps from the PBMC and BMMNC applied clustering analysis for the Functional FlowSOM (A) the Activation markers FlowSOM (B) and CIR FlowSOM (C).

Figure S9

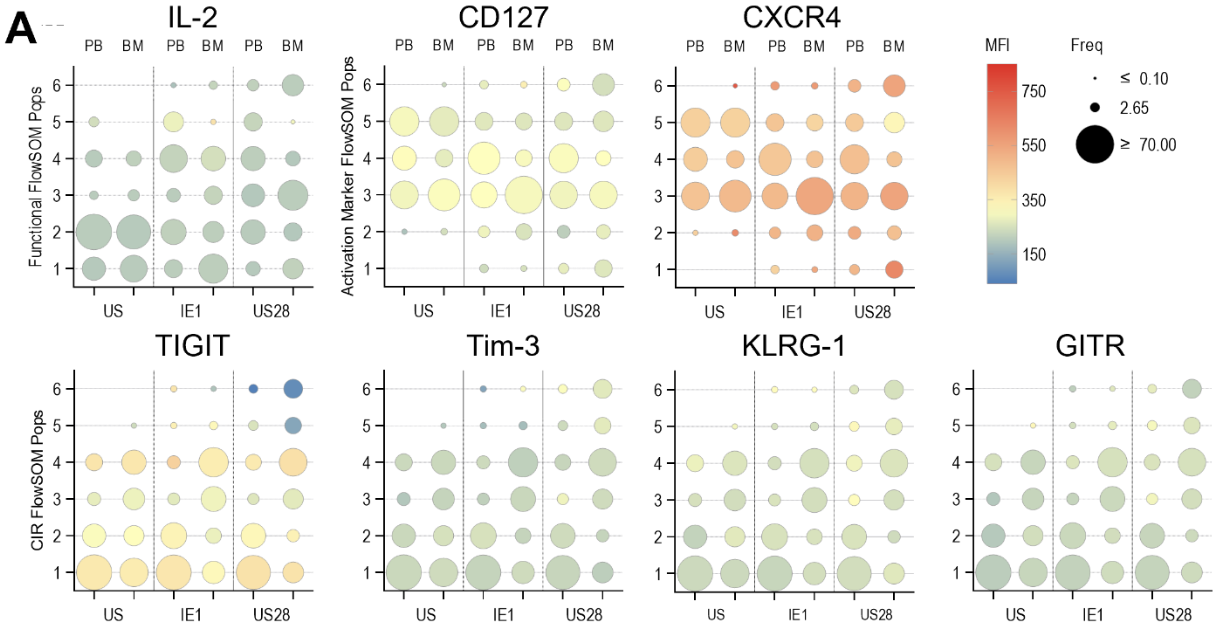

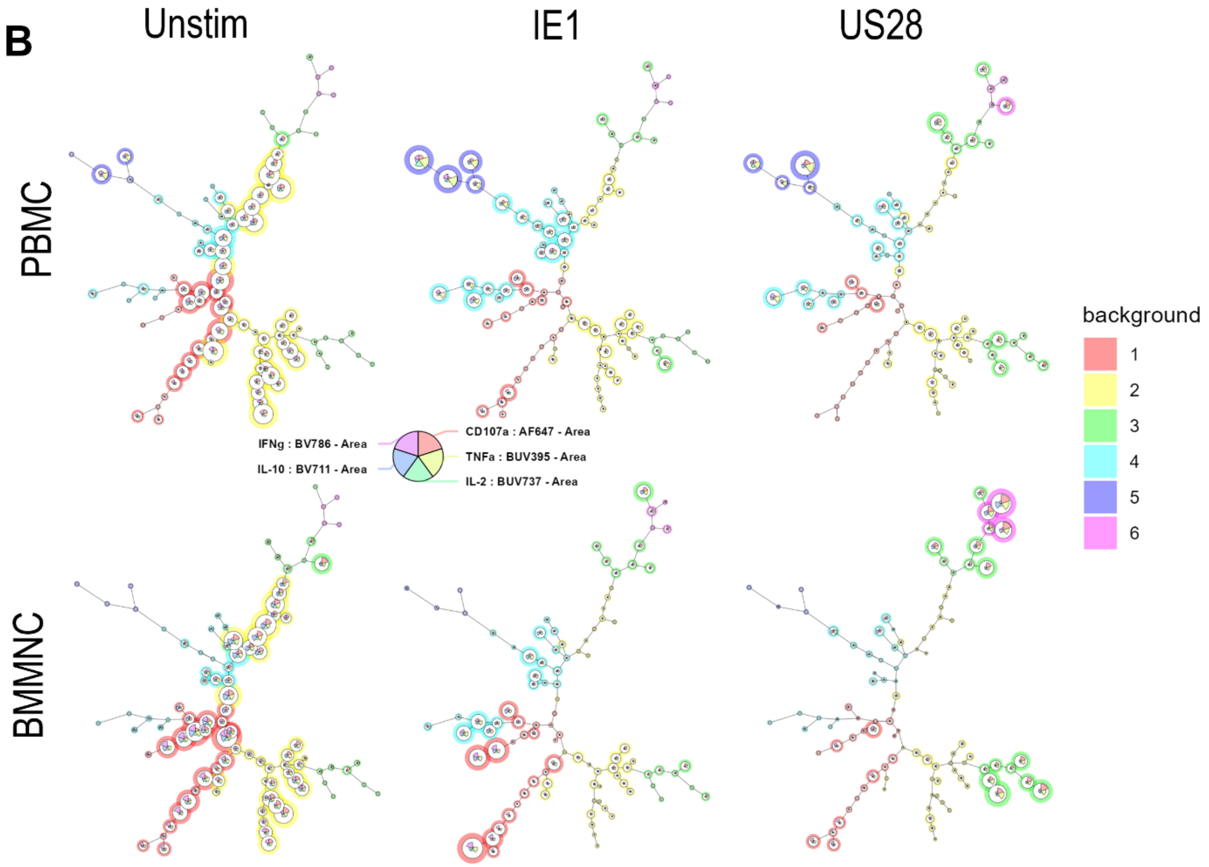
B
Unstim
IE1
US28
PBMC
BMMNC
IFNg : BV786 - Area
IL-10 : BV711 - Area
CD107a : AF647 - Area
TNFa : BUV395 - Area
IL-2 : BUV737 - Area
background
1
2
3
4
5
6

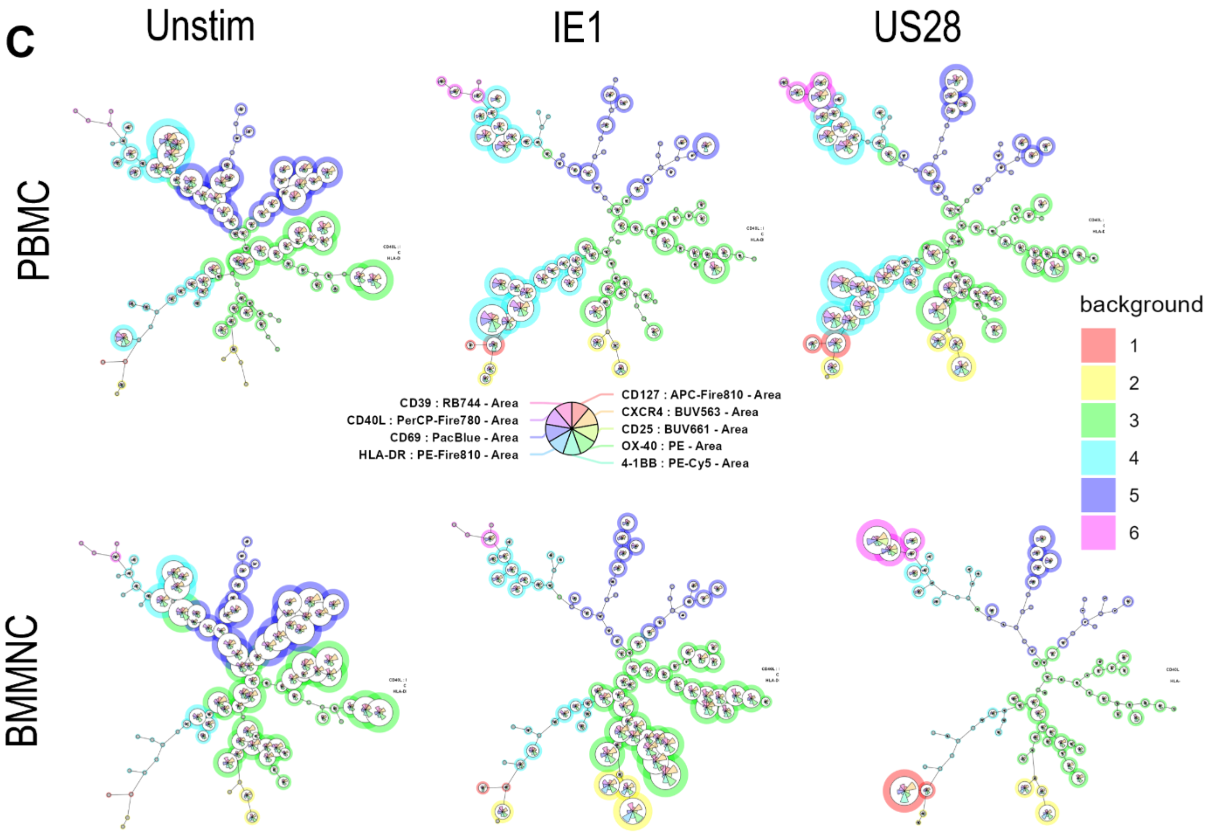
C
Unstim
IE1
US28
PBMC
BMMNC
CD39 : RB744 - Area
CD40L : PerCP-Fire780 - Area
CD69 : PacBlue - Area
HLA-DR : PE-Fire810 - Area
CD127 : APC-Fire810 - Area
CXCR4 : BUV563 - Area
CD25 : BUV661 - Area
OX-40 : PE - Area
4-1BB : PE-Cy5 - Area
background
1
2
3
4
5
6

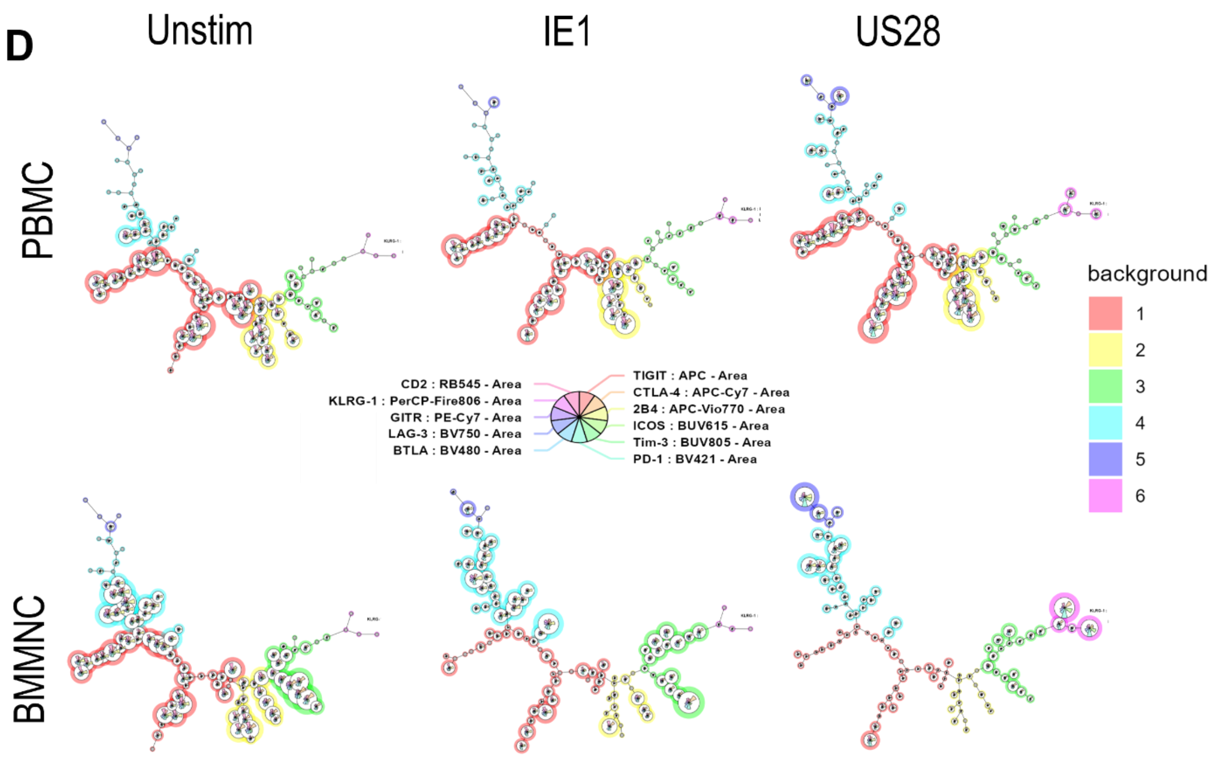

Supplementary Figure S9. Comparison of PBMC and BMMNC CMV specific CD4+ T cell analysis. HCMV specific T cells were identified and concatenated as described in the methods and Figures S3 and S4. Summarised are the remaining individual parameter bubble plots from the FlowSOM functional (IL-2), activation (CD127, CXCR4) and CIR (TIGIT, Tim-3, KLRG-1, GITR) clustering analyses (A). The individual Self-organising maps for each sample type and stimulation are shown for the Functional FlowSOM (B), Activation markers FlowSOM (C) and CIR FlowSOM (D).

Figure S10

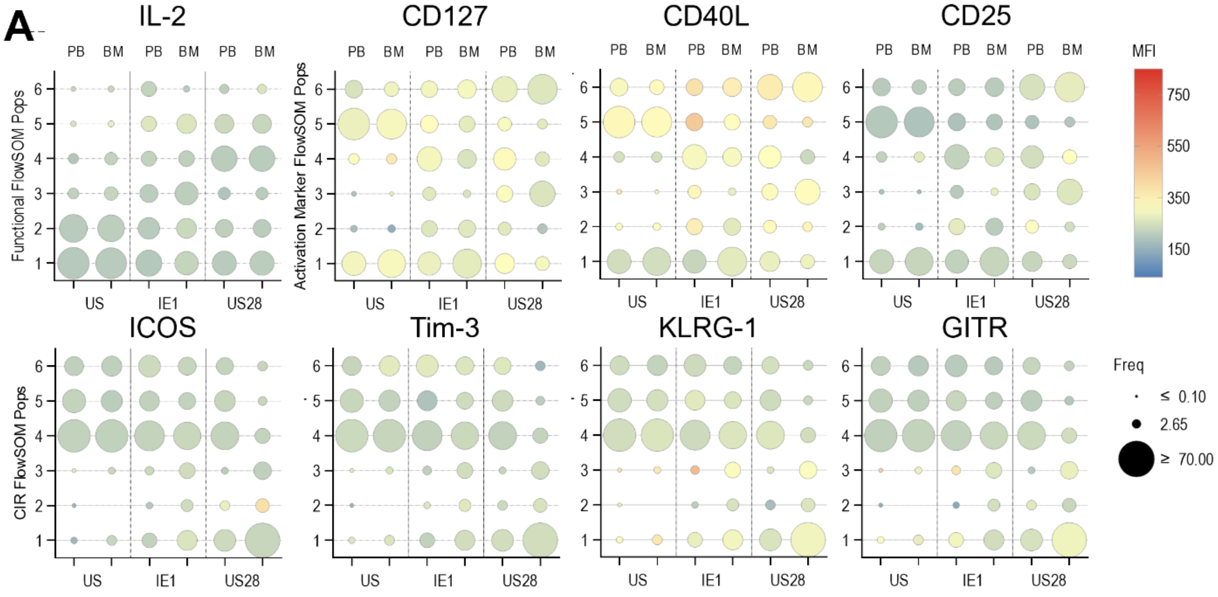

B

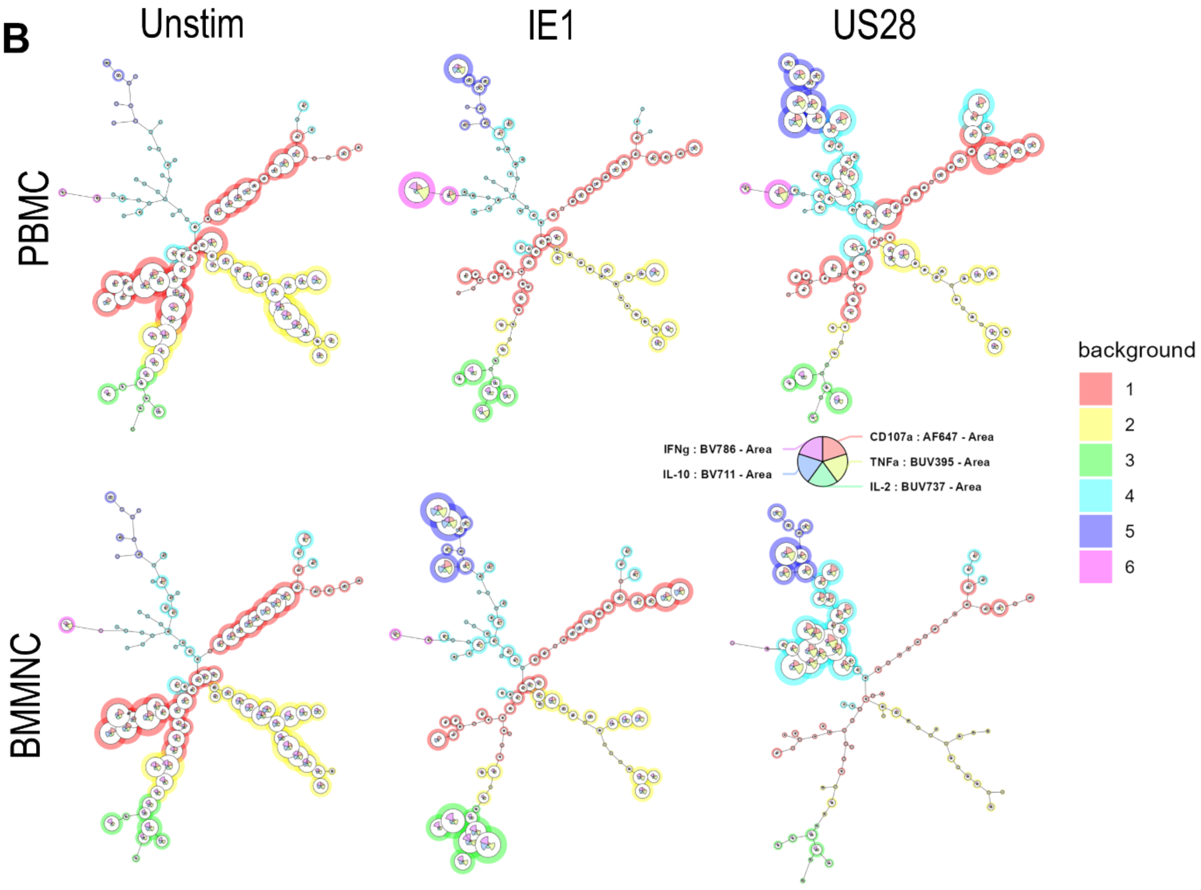
Unstim
IE1
US28
PBMC
BMMNC
IFNg : BV786 - Area
IL-10 : BV711 - Area
CD107a : AF647 - Area
TNFa : BUV395 - Area
IL-2 : BUV737 - Area
background
1
2
3
4
5
6

C

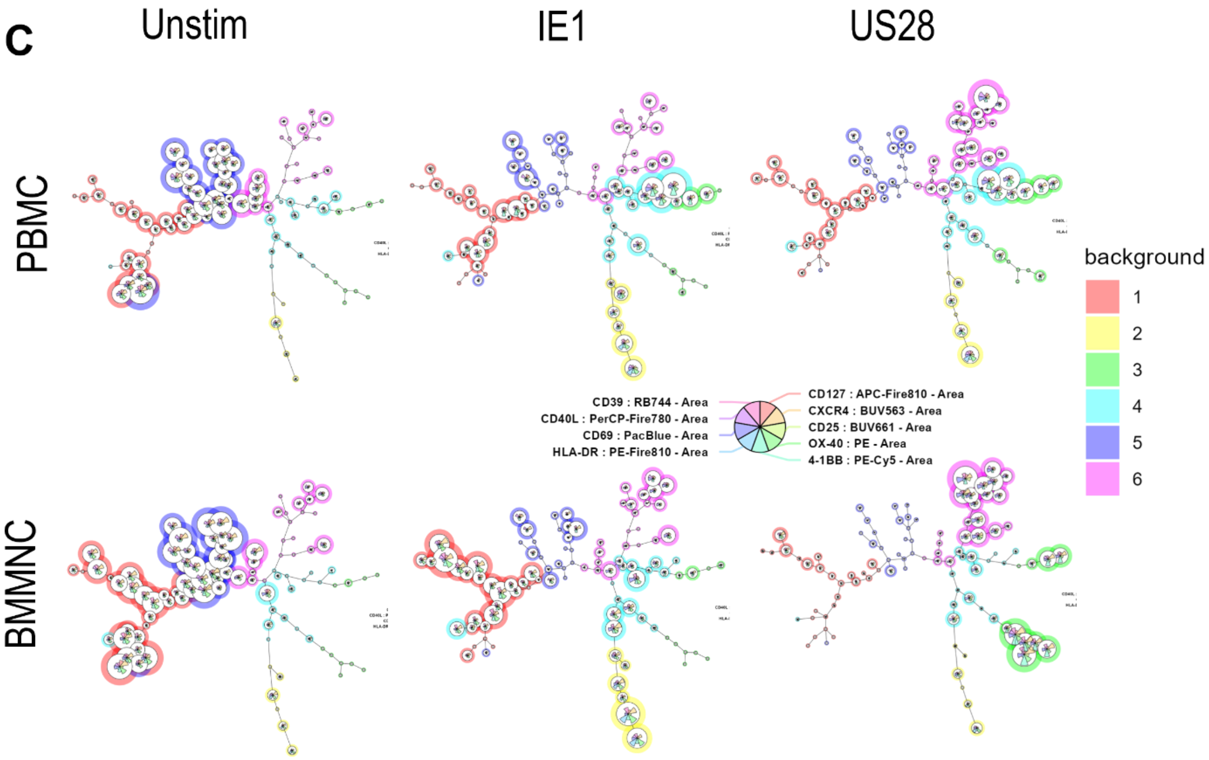
Unstim
IE1
US28
PBMC
BMMNC
CD39 : RB744 - Area
CD40L : PerCP-Fire780 - Area
CD69 : PacBlue - Area
HLA-DR : PE-Fire810 - Area
CD127 : APC-Fire810 - Area
CXCR4 : BUV563 - Area
CD25 : BUV661 - Area
OX-40 : PE - Area
4-1BB : PE-Cy5 - Area
background
1
2
3
4
5
6

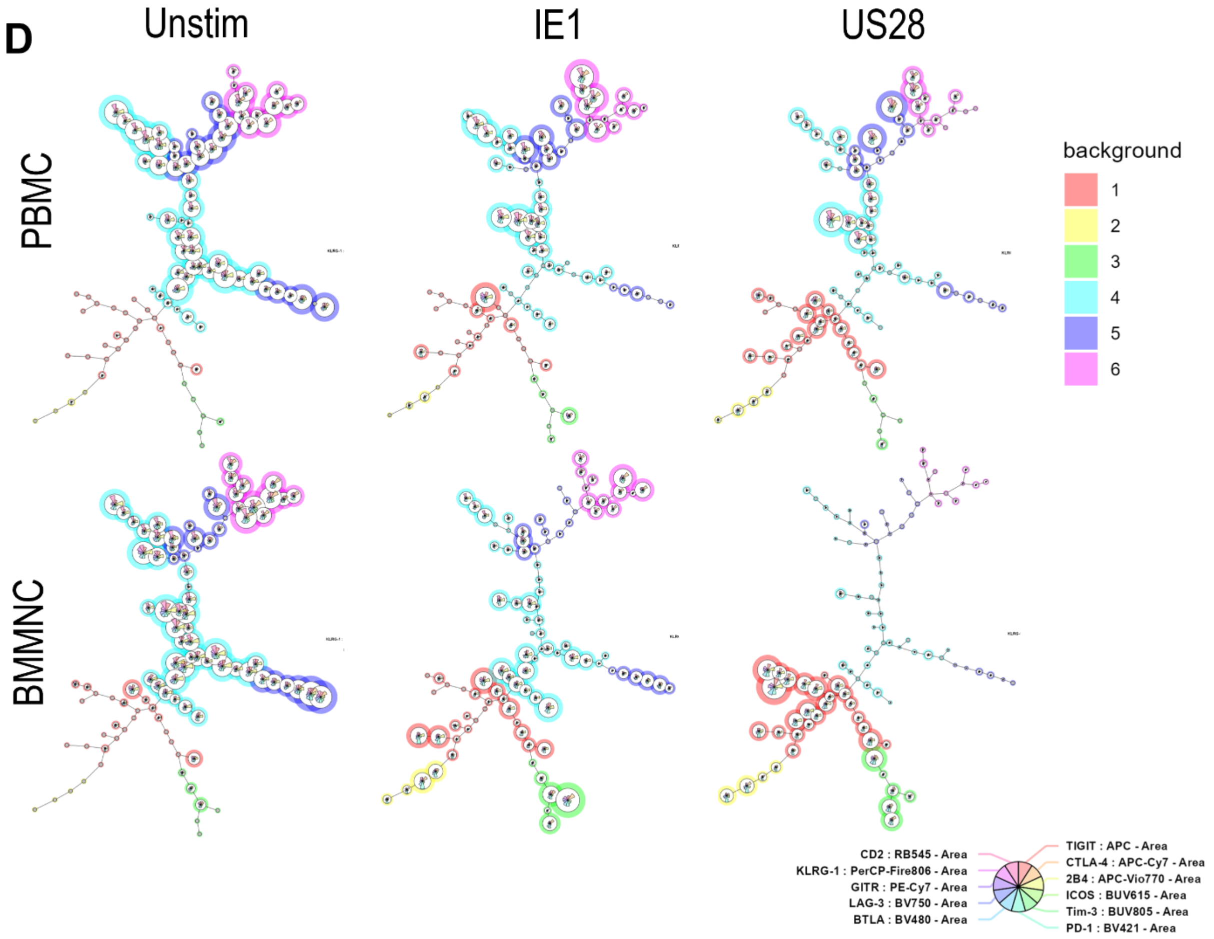

Supplementary Figure S10. Comparison of PBMC and BMMNC CMV specific CD8+ T cell analysis. HCMV specific T cells were identified and concatenated as previously described. Summarised are the remaining individual parameter bubble plots from the FlowSOM functional (IL-2), activation (CD127, CD40L, CD25) and CIR (ICOS, Tim-3, KLRG-1, GITR) clustering analyses (A). The individual Self-organising maps for each sample type and stimulation for the Functional FlowSOM (B), Activation markers FlowSOM (C) and CIR FlowSOM (D) are also shown.

Figure S11

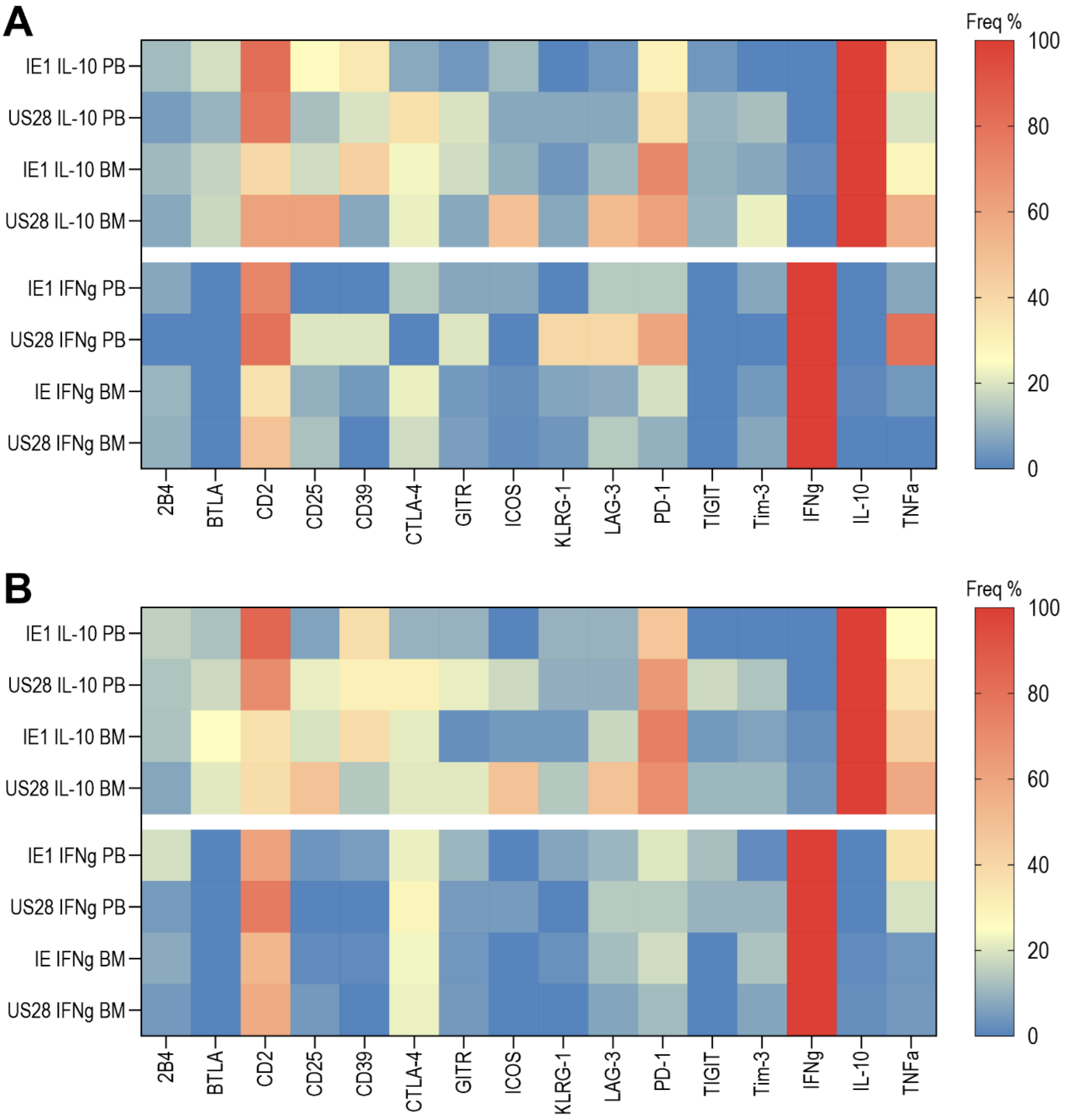

Supplementary Figure S11. Inhibitory Receptor expression on IL-10 secreting CMV specific T cells. The frequency of expression of 13 inhibitory or immunomodulatory associated proteins and cytokines on IE1 or US28 specific IL-10 secreting cells compared to IFNγ secreting cells are shown as a heatmap for CD4+ T cells (A) and CD8+ T cells (B).
